# Usp11 maintained the survival of marginal zone B cells under ionizing radiation by deubiquitinating DLL1 and JAG2

**DOI:** 10.1038/s41419-025-07377-7

**Published:** 2025-02-04

**Authors:** Jiaqi Sheng, Depei Wu, Jingzhe Shang, Xiaodan Fu, He Gao, Jianjie Rong, Jun Wang, Jiancheng Hu, Xiaofei Qi

**Affiliations:** 1 Department of Hematology, the First Affiliated Hospital of Soochow University & State Key Laboratory of Radiation Medicine and Protection, School of Radiation Medicine and Protection, Soochow University, Suzhou, 215000 China; 2 National Clinical Research Center for Hematologic Diseases, Jiangsu Institute of Hematology, Collaborative Innovation Center of Hematology, Suzhou, 215006 China; 3 Cyrus Tang Hematology Center & Institute of Blood and Marrow Transplantation, Suzhou, 215006 PR China; 4 State Key Laboratory of Common Mechanism Research for Major Diseases, Suzhou Institute of Systems Medicine, Chinese Academy of Medical Sciences & Peking Union Medical College, Suzhou, 215123 China; 5 Institutes of Biology and Medical Sciences, Suzhou Medical College of Soochow University, Suzhou, 215123 China; 6 Department of Vascular Surgery, Suzhou TCM Hospital Affiliated to Nanjing University of Chinese Medicine, Suzhou, 215000 China; 7 Laboratory of Molecular Mechanism & Targeted Therapy, National Cancer Center Singapore, Singapore General Hospital, Duke-NUS Medical School, Singapore, 168583 Singapore

**Keywords:** Immunopathogenesis, Immunogenetics

## Abstract

Efficacy of radiation therapy is compromised by hematopoietic and immune impairments, with elusive underlying causes. This study aimed to elucidate Usp11’s role in radiation-induced injuries and uncover related mechanisms. Utilized ARS mouse model to observe survival rates of Usp11^−/−^ (KO) mice post-TBI (Total Body Irradiation). Assessed lymphocyte and MZ B (Marginal Zone B) cell rates using histological analysis, single-cell sequencing, immunofluorescence (IF), immunohistochemistry (IHC), and flow cytometry (FCM). Conducted Co-IP and ubiquitination experiments for mechanism elucidation. Quantified IgM and IgG using ELISA and FC. Explored public databases for potential correlation molecules. Our findings indicated that Usp11^−/−^ mice exhibited improved survival rates following TBI, with the spleen playing a pivotal role. HE staining revealed a wider marginal zone in the spleen of Usp11^+/+^ mice post-irradiation. Single-cell sequencing, IF, IHC, and FCM analyses revealed a higher survival rate of MZ B cells in Usp11^−/−^ mice after irradiation. Furthermore, treatment with the Usp11 inhibitor, mitoxantrone, successfully targeted and inhibited Usp11, thereby alleviating the reduction in MZ B cells in the spleen following total body irradiation. Mechanistically, Usp11 sustained the survival of MZ B cells by regulating the ubiquitination of Notch’s ligands, DLL1 and JAG2, thereby promoting immune cell remodeling in the spleen. In conclusion, Usp11 played a crucial role in modulating immune system damage induced by ionizing radiation, primarily through ubiquitination of Notch ligands. This study provides insights into radiation-induced immune injuries and suggests Usp11 as a potential therapeutic target.

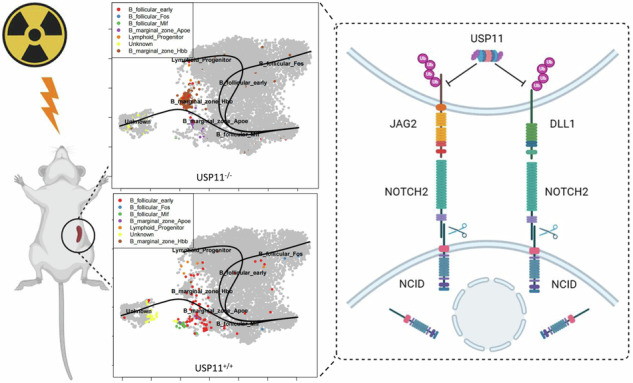

## Introduction

With the widespread use of radiation therapy in clinical practice, the related side effects of radiation are gradually increasing nowadays. Among numerous organs and tissues affected by radiation, hematopoietic and immune systems are extremely sensitive to it [1]. Systemic exposure to medium to high doses of ionizing radiation can lead to acute damage to the bone marrow and peripheral immune system, particularly affecting the innate immune system, and causing acute radiation sickness [2], This results in reduced resistance to infection and increased bleeding, leading to high mortality rates [3, 4]. Consequently, it becomes challenging for many patients to achieve ultimate success, posing a significant problem in clinical practice [5–7].

The spleen, a vital hematopoietic and immune organ primarily composed of lymphocytes, plays a pivotal role in maintaining the body’s immune function [8]. The immune cells present in the spleen mainly include T lymphocytes, B lymphocytes, and macrophages [9]. The function of B cells in the spleen is to produce antibodies, participate in immune responses, and contribute to the maintenance of immune system health. B cells in the spleen can be classified into B1 cells and B2 cells [10]. B2 cells can be further categorized into follicular B cells (FO B) and marginal zone cells (MZ B). MZ B cells are non-migratory and abundant in the marginal zone of the spleen [11, 12]. These cells can respond directly to blood-borne pathogens and then transform into antigen-presenting cells or IgM-producing plasma cells thus bridging the gap between the innate immune response and the adaptive immune response [13, 14]. Therefore, identifying the key molecules involved in the development, differentiation, and maintenance of MZ B cells is crucial for maintaining normal immune surveillance, immune defense, and immune homeostasis in the body [13, 15, 16].

The ubiquitin-proteasome pathway (UPS) constitutes a vital component within the extensive spectrum of cellular regulatory mechanisms, holding significance in B cell ontogeny, differentiation, apoptosis, and numerous other processes [17]. Deubiquitinating enzymes (DUBs), serving as essential regulators of ubiquitination, play a pivotal role in these intricate processes. Usp11, specifically functioning as a ubiquitin carboxy-terminal hydrolase, exerts regulatory influence over the stability and functionality of proteins such as TGF-β receptors, IAP, γH2AX, and NF-κB [18, 19], which are implicated in inflammatory and immune responses. The regulation of Usp11 turnover can contribute to retinal degeneration, and it exhibits a dual function in the context of cancers [19]. Our prior research revealed the ablation of Usp11 in bone marrow cells demonstrated a profound impact on delaying the onset of acute graft-versus-host disease (aGvHD). Moreover, the deletion of Usp11 in splenic tissues was also contributed to this phenomenon. Based on these observations, we propose a hypothesis that the spleen may play a pivotal role in the immune regulation mediated by Usp11 [18]. In this paper, using mouse models, we explored the role of Usp11 in the maintenance of MZ B cells and elucidated the possibility of Usp11 serving as an effective intervention target.

## Results

### Usp11 knockout alleviated the radiation-induced damage

**Fig. 1:**
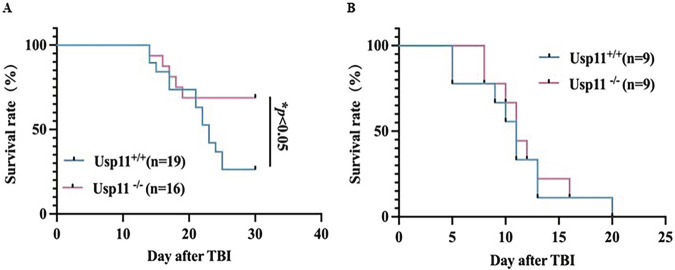
Usp11 knockout alleviated the radiation-induced damage. **A** Survival curve of Usp11^+/+^ and Usp11^−/−^ mice after whole-body 7.5 Gy irradiation. *p < 0.05. **B** Survival curve of Usp11^+/+^ and Usp11^−/−^ splenectomy mice after whole-body irradiation.

After exposing mice to whole-body irradiation at a dose of 7.5 Gy, we established an acute radiation syndrome (ARS) mouse model. With this model, we conduct a comprehensive study to thoroughly explore the consequences of radiation exposure on the physiology and pathophysiology of Usp11^−/−^ and Usp11^+/+^ mice. As results shown, following 7.5 Gy irradiation, Usp11^−/−^ mice exhibited faster weight recovery (Supplementary Fig. S1A), better survival status (Supplementary Fig. S1B) and a higher survival rate compared to wild-type mice (Fig. 1A).

The histological examination of bone marrow revealed significant architectural alterations in both irradiated Usp11^+/+^ mice and Usp11^−/−^ mice. It was observed that the bone marrow of both groups sustained severe damage, with only a limited number of hematopoietic cells remaining and adipose tissue occupying a significant portion of the bone marrow space, no significant difference in femur injury was observed between the two groups at day 6 or 12 post-TBI (Supplementary Fig. S2A and B), prompting the change of bone marrow was not the key factor that contributes to the prolongation of survival.

Subsequently, we focused on another important hematopoietic and immune organ, the spleen. The survival rates were evaluated of the two groups following spleen resection and whole-body irradiation with 7.5 Gy. Interestingly, the survival rates of both groups became comparable (Fig. 1B).

Collectively, these findings suggest that Usp11 played a role in promoting radiation-induced damage, particularly in the spleen, which appeared to be a critical organ in this process.

### Usp11 regulated ionizing radiation-induced remodeling of immune cells in spleen

**Fig. 2:**
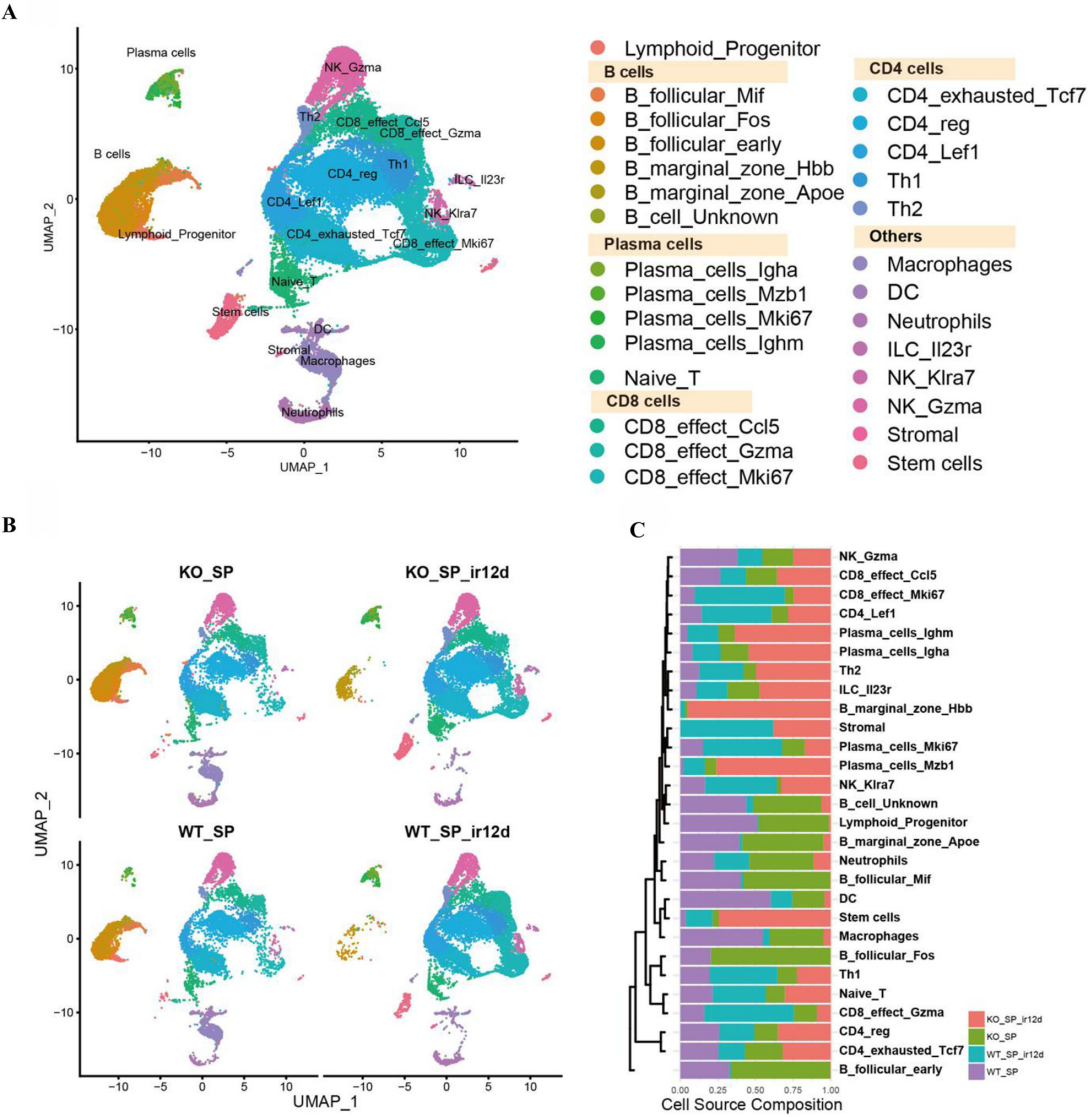
The characteristics of different cell groups of mice spleen. **A** The diverse immune cell landscape of the mouse spleen. Different types of cells are labeled with different colors. **B** Immune cells profiles of the mice spleen on day 0, 12 after TBI. WT_SP spleen of Usp11^+/+^ mice on day 0 post-TBI, WT_SP_ir12d spleen of Usp11^+/+^ mice on day 12 post-TBI, KO_SP spleen of Usp11^−/−^ mice on day 0 post-TBI, KO_SP_ir12d spleen of Usp11^−/−^ mice on day 12 post-TBI. **C** Trends of different immune cells in the spleen of mice. WT_SP spleen of Usp11^+/+^ mice on day 0 post-TBI, WT_SP_ir12d spleen of Usp11^+/+^ mice on day 12 post-TBI, KO_SP spleen of Usp11^−/−^ mice on day 0 post-TBI, KO_SP_ir12d spleen of Usp11^−/−^ mice on day 12 post-TBI.

In order to discover the characteristic changes in spleen cells of Usp11^+/+^ mice and Usp11^−/−^ mice before and after irradiation, we conducted single-cell sequencing. After rigorous quality control, we isolated a total of 50,485 immune cells across four distinct samples, categorized into 28 unique clusters (Fig. 2A). The uniform manifold approximation and projection (UMAP) analysis highlights the diverse immune cell landscape. Results showed that mouse spleen immune cells were reconstruct in Usp11^−/−^ mice, more prominent after radiation exposure, with more B cells in the Usp11^−/−^ sample (KO) compared to the wild-type (WT) (Fig. 2B and C). This differential numbers and response under stress conditions underscore the pivotal role of B cells in modulating immune responses within these settings. The significant shift of the immune cell composition, suggesting a crucial involvement of B cells in the immune architecture and response dynamics post-irradiation (Fig. 2C).

### Usp11 modulated histological alterations of the spleen that were triggered by ionizing radiation exposure

To further validate the results of single-cell sequencing, we performed HE staining on the mouse spleen. Histological examination also revealed significant alterations in spleen in both experimental groups. These alterations were characterized by an indistinct splenic structure, accompanied by a decrease in lymphocyte counts. Notably, diffuse exudation of red blood cells was observed throughout the splenic parenchyma, indicating extensive cellular infiltration. Mild extramedullary hematopoiesis, a compensatory process for bone marrow dysfunction, was also observed.

**Fig. 3:**
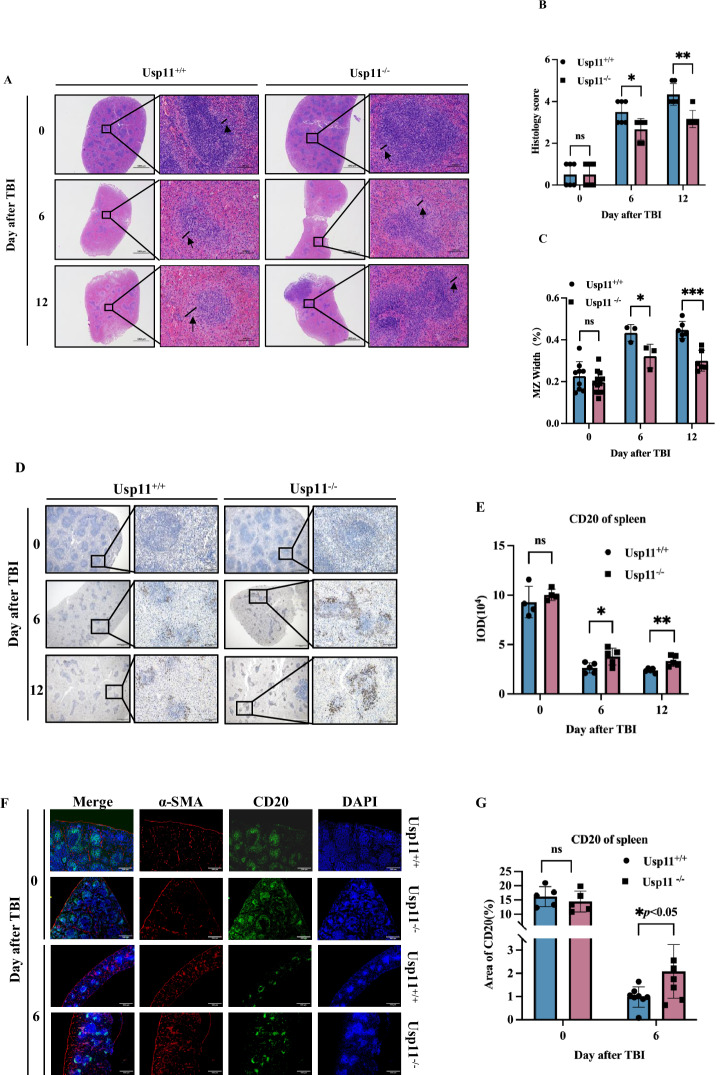
Usp11 modulated histological alterations of the spleen that were triggered by ionizing radiation exposure. **A** Histological examination of mice spleens on days 0, 6 and 12 after TBI (*n* = 3). **B** Histology score of mice spleens on days 0, 6 and 12 after TBI (*n* = 3), *p < 0.05, **p < 0.01. **C** The ratio of the width of the marginal zone of the spleen between Usp11^+/+^ and Usp11^−/−^ mice on days 0, 6 and 12 after TBI (*n* = 3). The ratio was obtained from the percentage of the width of the marginal zone to the overall width of the white pulp. *p < 0.05, ***p < 0.005. **D** Immunohistochemistry examination of CD20^+^ in the spleen of Usp11^+/+^ *vs* Usp11^−/−^ mice on days 0, 6, and 12 after 7.5 Gy TBI. **E** Statistical analysis of CD20 immunohistochemical IOD values in mouse spleen. *p < 0.05, **p < 0.01. **F** Immunofluorescence examination of CD20^+^ in the spleen of Usp11^+/+^ *vs* Usp11^−/−^ mice on days 0 and 6 after 7.5 Gy TBI. **G** Statistical analysis of CD20 immunofluorescence proportion in mouse spleen. *p < 0.05, **p < 0.01.

In a comparative analysis of the spleens from Usp11^−/−^ mice and their Usp11 wild-type counterparts, several key differences were observed. Notably, the Usp11^−/−^ mice displayed a decrease in their histology score post TBI (Fig. 3A, B), indicating alterations in cellular turnover and survival. Additionally, a significant narrowing of the marginal zone of the spleen was evident in the Usp11^−/−^ mice post TBI (Fig. 3A, C).

To further investigate the cellular mechanisms underlying these histological alterations, we conducted immunohistochemical and immunofluorescence staining analysis of spleens harvested from female Usp11^+/+^ and Usp11^−/−^ mice at various time points post-TBI. As we know, the marginal zone, a crucial region for immune surveillance and cell trafficking, is populated by macrophages, MZ B cells, and T cells.

Utilizing CD169 antibody, a specific marker for macrophages, macrophage numbers in both groups of mice were all significantly decreased within the spleen at both day 6 and day 12 post-TBI (Supplementary Fig. S3A and B). Employing immunofluorescence staining no significant difference were declared in the distribution of macrophages in the marginal zone of the spleen between the two groups, whether at baseline or at day 6 post-TBI (Supplementary Fig. S3C and D).

With CD3 antibody, a specific marker for T cells, we undertook a detailed analysis of T cell populations and their spatial distribution within the marginal zone of the spleen in two groups, both before and after TBI. Our immunohistochemical (Supplementary Fig. S4A and B) and immunofluorescence staining (Supplementary Fig. S4C and D) analyses consistently demonstrated no significant differences in T cell counts or their distribution patterns within the marginal zone of the spleen across both groups, regardless of whether the mice were assessed at baseline or at multiple time points following TBI.

Subsequently, we conducted CD20 staining to examine the alterations in B-cell populations in the spleens of two groups of mice. The results demonstrated that at baseline, the distribution of B cells was comparable in the spleens of both groups. However, on days 6 and 12 post-TBI, we observed a higher number of B cells left in the spleens of Usp11^−/−^ mice compared to their Usp11^+/+^ counterparts (Fig. 3D, E). Additionally, immunofluorescence staining revealed a marked increase in B cells at day 6 post-TBI, particularly within the marginal zone of the spleen (Fig. 3F, G).

These observations provide valuable insights into the effects of the absence of the Usp11 gene on the distribution of B cells in the spleen post-TBI, which may be the reason for the relatively narrower marginal area of the spleen after TBI in mice after Usp11 knockout.

To validate this hypothesis, we compiled and analyzed published data. Through interrogation of the GDS2762 database, we observed a significant down-regulation of Usp11 expression in B cells following activation by IFN-β treatment (Supplementary Fig. S5A). Furthermore, utilizing the GDS2554 database, we found that the expression of Usp11 in B cells derived from aggressive lymphoma samples was significantly reduced compared to that in normal resting B cells (Supplementary Fig. S5B).

These findings suggest that Usp11 may have a pivotal role in regulating B lymphocyte-associated biological processes.

### Usp11 sustained the survival of MZ B post-TBI

**Fig. 4:**
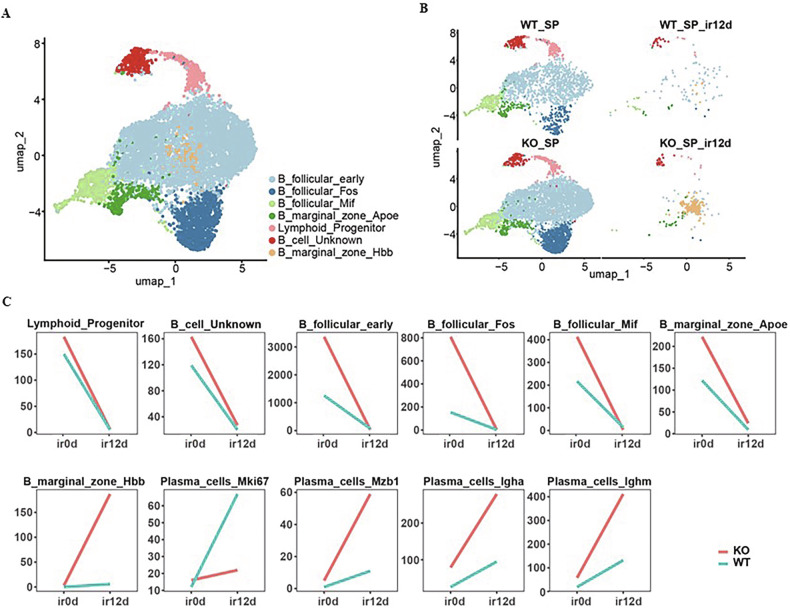
The characteristics of B cell groups of mice spleen. **A** B cell landscape of the mouse spleen. Different types of cells are labeled with different colors. **B** B-cell subsets of the mice spleen on day 0, 12 after TBI. WT_SP spleen of Usp11^+/+^ mice on day 0 post-TBI, WT_SP_ir12d spleen of Usp11^+/+^ mice on day 12 post-TBI, KO_SP spleen of Usp11^−/−^ mice on day 0 post-TBI, KO_SP_ir12d spleen of Usp11^−/−^ mice on day 12 post-TBI. **C** Trends of B cells in the spleen of mice. WT_SP spleen of Usp11^+/+^ mice on day 0 post-TBI, WT_SP_ir12d spleen of Usp11^+/+^ mice on day 12 post-TBI, KO_SP: spleen of Usp11^−/−^ mice on day 0 post-TBI, KO_SP_ir12d spleen of Usp11^−/−^ mice on day 12 post-TBI.

To elucidate the dynamics of different B cell subtypes in response to genetic and environmental alterations in our single cell sequencing results, we meticulously segregated and analyzed seven distinct B cell subclusters, excluding plasma cells (Fig. 4A). Usp11 was detectable in all these B cell subclusters (Supplementary Fig. S6). These subclusters encompass three follicular B cell groups and two marginal zone clusters. Notably, all three follicular clusters showed an upsurge in the Usp11^−/−^ (KO) samples, suggesting a robust response to knockout conditions. But after irradiation, there was no difference between the two groups due to the death of most cells. Contrastingly, the two marginal zone clusters exhibited divergent trends. Specifically, the B_marginal_zone_Hbb cluster demonstrated a clearly enriched in the Usp11^−/−^ samples post-irradiation (Fig. 4B, C, Supplementary Fig. S7A and B).

**Fig. 5:**
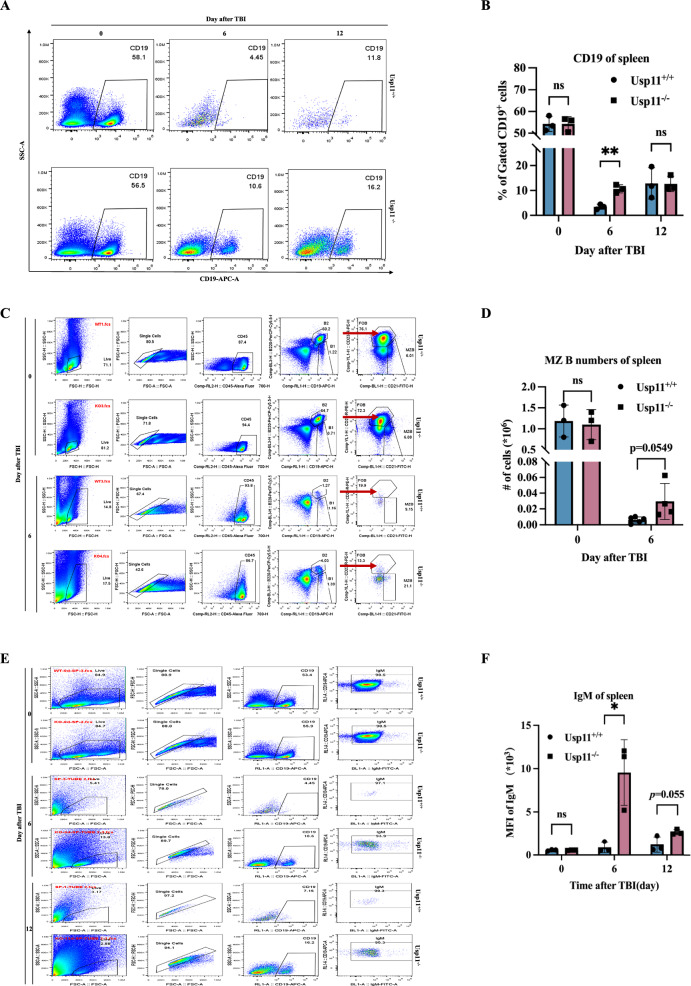
Usp11 sustained the survival of MZ B post-TBI. **A** The changes of CD19^+^ lymphocytes in the spleen of Usp11^+/+^ and Usp11^−/−^ mice at 0, 6, and 12 days after TBI by flow cytometry detection. **B** Statistical analysis of flow cytometry detection. **C** MZ B cells in the spleen of Usp11^+/+^ and Usp11^−/−^ mice on days 0 and 6 after TBI. **D** Statistical analysis of MZ B flow cytometry detection in mouse spleen. **E** IgM in the spleen of Usp11^+/+^ and Usp11^−/−^ mice at 0, 6, and 12 days after TBI. **F** Statistical analysis of IgM in mouse spleen. *p < 0.05, **p < 0.01.

Next, the splenic B lymphocyte populations in Usp11^+/+^ and Usp11^−/−^ mice were analyzed *via* flow cytometry for CD19 expression on days 0, 6, and 12 post-TBI. Notably, no significant differences in the baseline levels of B lymphocytes were observed between the two groups. Post-TBI, a reduction in splenic B lymphocyte counts was evident in both groups, albeit with a higher number of B lymphocytes in Usp11^−/−^ mice compared to Usp11^+/+^ mice on day 6 (Fig. 5A, B). Conversely, no significant differences were noted in the splenic T cells and macrophage cells between Usp11^−/−^ and Usp11^+/+^ mice on day 6 post-TBI (Supplementary Fig. S8)

Furthermore, splenic cells were cultured in vitro. After exposure to 1 Gy radiation, more γH_2_AX-positive cells were present within the splenic B lymphocyte populations of Usp11^+/+^ mice, irrespective of whether they belonged to the follicular (FO) B cell subset or the marginal zone (MZ) B cell subset, at both 2 and 6 h post-irradiation (Supplementary Fig. S9).

To further investigate the differences in marginal zone B (MZ B) cells between female Usp11^+/+^ and Usp11^−/−^ mice, flow cytometry was employed for additional staining and sorting on days 0 and 6 post-TBI. Statistical analysis revealed no significant differences in the baseline levels of MZ B cells between the groups. Following TBI, a decrease in the overall MZ B cell population was observed in both mouse groups, with Usp11^−/−^ mice exhibiting a higher number of MZ B cells post-TBI (Fig. 5C, D).

MZ B cells function similarly to B1 cells and produce antibodies to the IgM isotype. The spleen and serum of Usp11^+/+^ and Usp11^−/−^ mice on days 0, 6, and 12 post-TBI will be taken for spleen IgM flow cytometry detection, and ELISA will be used to detect the changes of IgM and IgG antibodies in serum. Notably, no significant differences in baseline IgM levels were observed between the two groups in both spleen and serum. However, on day 6 post-TBI, Usp11^−/−^ mice exhibited higher IgM antibody production in the spleen compared to Usp11^+/+^ mice (Fig. 5E, F). Additionally, Usp11^−/−^ mice serum displayed higher IgM levels and lower IgG antibody levels on day 12 post-TBI (Supplementary Fig. S10A and B).

These findings align with the results obtained from immunohistochemistry and single-cell analyses.

### Intervention strategies targeting Usp11 to modulate immune system damage induced by ionizing radiation exposure

**Fig. 6:**
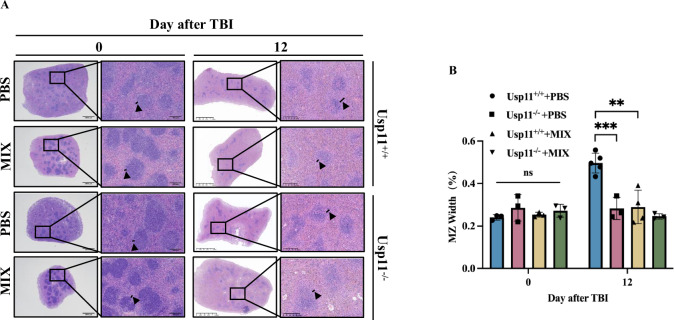
MIX simulated the morphological changes of the Usp11^−/−^ mice spleen post-TBI. **A** Comparison of morphological changes of the Usp11^−/−^ mice spleen on days 0 and 12 post-TBI with or without MIX intervention. **B** Comparative analysis of the width of the marginal zone of the spleen between Usp11^+/+^ and Usp11^−/−^ mice after MIX intervention. **p < 0.01, ***p < 0.001.

**Fig. 7:**
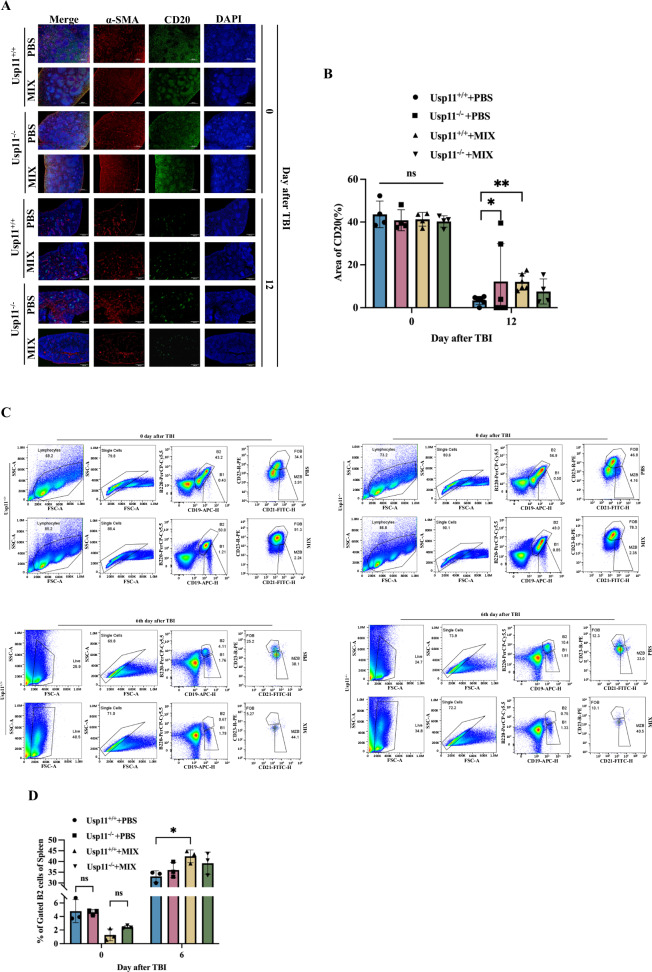
Intervention strategies targeting Usp11 modulated immune system damage induced by ionizing radiation exposure. **A** Comparative analysis of B lymphocyte changes in CD20^+^ in the spleen of female Usp11^+/+^ *vs* Usp11^−/−^ mice with or without MIX treated. **B** Statistical analysis of immunofluorescence staining of spleen CD20. **C** MZ B cell changes in Usp11^+/+^ *vs* Usp11^−/−^ mice on days 0 and 6 post- TBI after MIX treated. **D** Statistical analysis of MZ B in Usp11^+/+^ *vs* Usp11^−/−^ mice after MIX treated. *p < 0.05, **p < 0.01.

Mitoxantrone (MIX), an FDA-approved anticancer drug, had demonstrated the ability to modulate the expression of intracellular Usp11 in diverse cell models and was recognized as an inhibitor of Usp11 [20, 21]. To validate the role of Usp11 in sustaining the survival of marginal zone B (MZ B) cells, wild-type mice were administered mitoxantrone three days, followed by TBI. HE staining was performed to analyze and compare the morphological changes in the spleen and the ratio of splenic marginal zone width in these mice. The results revealed a narrowing of the splenic marginal zone in Usp11^+/+^ mice after MIX intervention (Fig. 6A, B). Fluorescence staining for CD20 indicated that targeted mitoxantrone intervention mitigated the reduction in MZ B cells in wild-type mice following ionizing radiation (Fig. 7A, B). Similar with our previous results, no significant difference in CD169^+^ (Supplementary Fig. S11A and B) and CD3^+^ cells (Supplementary Fig. S12 A and B) with or without MIX-treated post-TBI.

Subsequent flow cytometry analysis demonstrated that, consistent with previous findings, the total percentage of MZ B cells in Usp11^+/+^ mice exposed to TBI presented an upward trend and treated with MIX was significantly higher than in those treated with PBS (Fig. 7C, D). And serum IgM levels in these mice on days 0 and 6 post-irradiation also showed that MIX intervention reduced the shift of antibody types, with a more pronounced effect observed on day 6 post-TBI (Supplementary Fig. S13).

### Usp11 sustained the survival of MZ B cells by regulating the ubiquitination of Notch’s ligands

As we known, the Notch signaling pathway, particularly the Notch ligands DLL1 and JAG2, as well as their active fragments, were integral in sustaining the survival and proliferation of MZ B cells [22–24]. Blocking this pathway results in a diminution in the MZ B cell population [24]. To elucidate the underlying mechanism of Usp11’s influence on MZ B cell remodeling following radiation exposure, we investigated a potential correlation between Usp11 and DLL1, JAG2 using the GTEx and TCGA databases. Our analysis revealed a positive correlation between Usp11 expression in normal whole blood and spleen with the expression of DLL1 (Supplementary Fig. S14A) and JAG2 (Supplementary Fig. S14C). Similarly, in the AML database, Usp11 exhibited an association with DLL1 and JAG2 (Supplementary Fig. S14B and D). A comparable trend was observed in the DLBC (diffuse large B-cell lymphoma) dataset, where Usp11 and JAG2 displayed a correlation (Supplementary Fig. S14E). These findings indicate a potential interplay between Usp11 and DLL1 or JAG2 in immune organs and related diseases.

**Fig. 8:**
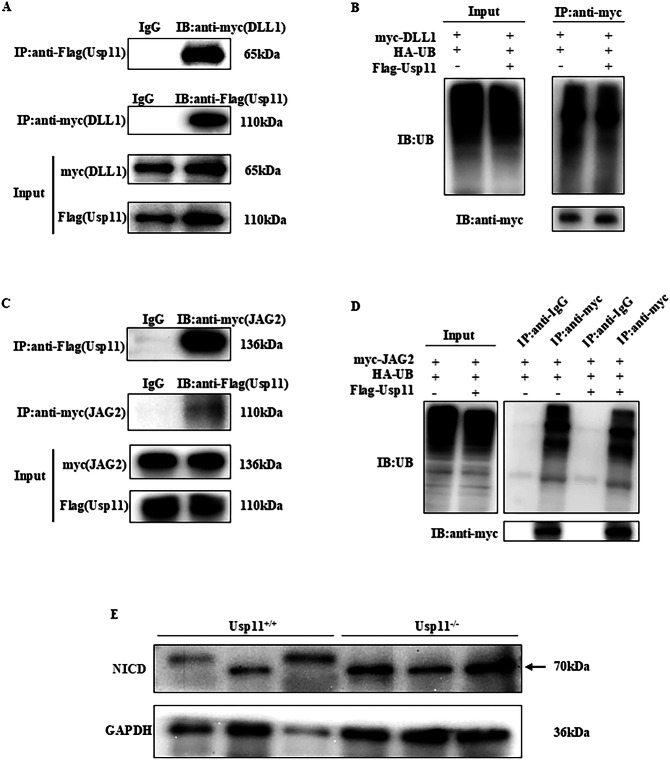
Usp11 regulated the ubiquitination of Notch’s ligands. **A** Usp11 interacted with DLL1. Flag-tagged Usp11, and myc-tagged DLL1 were co-transfected into HEK293T cells for 72 h. Cell lysates were precipitated with anti-myc or anti-Flag beads followed by immunoblotting. Total cell lysate (Input) were used as controls. **B** The 293T cells used to detect ubiquitination were overexpressed with Ub and DLL1, and two groups were set up to overexpress Usp11 and unloaded, using myc-tagged antibody pulldown proteins. **C** Usp11 interacted with JAG2. Total cell lysate (Input) and IgG were used as controls. **D** Usp11 inhibited IL-6 ubiquitination. The 293T cells were overexpressed with Ub and JAG2, and overexpressed Usp11 and unloaded were set up in two groups, using myc-tagged antibody pulldown proteins. **E** Comparison of NICD expression in spleen cells of Usp^11+/+^ with Usp11^−/−^ mice on day 6 post 7.5 Gy TBI.

Subsequently, co-immunoprecipitation (Co-IP) and ubiquitination experiments were performed on the Notch ligands DLL1 and JAG2 with Usp11. These experiments revealed a robust interaction between DLL1 and JAG2 with Usp11 (Fig. 8A, C). Similar results were observed in mouse spleen tissue (Supplementary Fig. S15). Furthermore, Usp11 inhibited the ubiquitination of DLL1 and JAG2 (Fig. 8B, D; Supplementary Fig. S16). Consistent with these findings, the splenic expression level of the Notch intracellular domain (NICD) was higher in Usp11^−/−^ mice compared to Usp11^+/+^ mice (Fig. 8E)

## Discussion

Addressing the deleterious effects of moderate to high doses of radiation on the hematopoietic and immune systems, which compromises the body’s immune defenses and elevates the innate immune response, continues to pose a significant challenge in the field of radiation protection [2]. Unfortunately, current therapeutic options for mitigating radiation-induced hematopoietic and immune systems damage are limited, primarily due to the lack of identified specific molecular targets for protective research. Moreover, despite extensive research efforts spanning several years, effective medical protection measures that are devoid of adverse side effects have yet to be discovered [4, 25].

The hematopoietic and immune systems exhibit remarkable sensitivity to ionizing radiation, with its acute response primarily manifesting in the diminished numbers of lymphocytes and immune-active cells, suppression of antibody shifting, and ultimately culminating in immunodeficiency. This immunodeficiency can trigger a cascade of complications [5–7]. As a pivotal immune organ, the spleen harbors immune cells that exhibit a precise hierarchical and spatial organization, spanning from the outer to inner regions. These cells include macrophages, MZ B cells, follicular B (FO B) cells, and T cells [9].

Ubiquitination, a crucial post-translational modification process for proteins, has been firmly established as the primary signaling mechanism regulating inflammation and immune response. This process plays a pivotal role in modulating immune responses via the ubiquitin-proteasome system (UPS) [26]. In the context of B cell biology, ubiquitination has been shown to significantly influence B cell development [27], differentiation [28], survival, apoptosis [29] signal transduction, and various other biological processes [17, 30–33].

Notably, Notch signaling is a decisive factor in dictating the fate of B cells, particularly for marginal zone B (MZ B) cells [24]. Deficiencies in components of the Notch signaling pathway, such as Notch2, Delta-like 1 (DLL1) [22, 34], and JAG2 [23], have been observed to impact the survival and development of MZ B cells, whereas they have no significant effect on follicular B (FO B) cells [34].

The Notch receptor on the cell membrane undergoes ubiquitination-mediated endocytosis, resulting in its internalization into the endosome. Inside the endosome, the Notch receptor can be recycled back to the cell membrane, cleaved into its active form NICD (Notch Intracellular Domain), or transported to the lysosome for degradation. The ubiquitination of the ligand triggers its endocytosis, which exerts a pulling force on the bound receptor, leading to the extension of the NRR (Notch Receptor Region) domain and exposing the S2 cleavage site. Subsequent to S2 cleavage, the remaining portion of the Notch receptor is referred to as NEXT. NEXT can be further processed at the cell membrane by γ-secretase or internalized into endosomes, ultimately resulting in its cleavage into NICD [24]. Consequently, an up-regulation in ligand ubiquitination results in an increased production of NICD, thereby amplifying the signals that promote the survival, proliferation, and differentiation of MZ B cells.

In this study, we observed an extended survival rate among Usp11^−/−^ mice following irradiation, with a pivotal role attributed to the spleen. Utilizing HE staining, we detected a significant difference in the marginal zone width between Usp11^+/+^ and Usp11^−/−^ mice post-irradiation. Further investigations through single-cell sequencing, immunofluorescence, immunohistochemistry, and flow cytometry revealed a greater survival rate of MZ B of the spleen in Usp11^−/−^ mice after irradiation. Our findings revealed that in the control group, the proportion of B cells declined on day 6 post-total body irradiation (TBI) and subsequently recovered by day 12. Conversely, the Usp11 knockout (KO) group did not exhibit such fluctuations within the 6–12day period. Although the precise underlying mechanism remains elusive, the increased cellular resistance to radiation following Usp11 knockout may be a significant contributing factor.

Furthermore, our results found that IgM levels could be 10-fold higher in irradiated Usp11 KO spleens compared to non-irradiated KO spleens while MZ B cell numbers decreased. This phenomenon occurred maybe because irradiation induced widespread cell apoptosis, releasing self-antigens that were recognized by MZ B cells. The recognition of these antigens activated the MZ B cells, resulting in a notable increase in IgM expression. As a result, IgM levels significantly rose in KO mice, despite the decline in MZ B cell count. However, in Usp11 WT mice, which were not resistant to radiation, the number of MZ B cells decreased to critically low levels after irradiation, rendering this effect unobservable.

To further elucidate the role of Usp11, we employed the Usp11 inhibitor, mitoxantrone, which successfully targeted and inhibited Usp11, subsequently alleviating the reduction in MZ B cells in the spleen following TBI. Additionally, we discovered that Usp11 sustains the survival of MZ B cells by regulating the ubiquitination of Notch’s ligands.

The limitations of this study encompass several aspects. Firstly, the absence of an ideal antibody hindered our ability to conduct experiments utilizing IP-UB with anti-DLL1 or anti-JAG2 antibodies, which would have verified DLL1 and JAG2 as deubiquitination substrates of Usp11. Secondly, we did not investigate the impact of Usp11 over-expression or knockdown on DLL1 and JAG2 protein levels. Lastly, no rescue experiments were performed to bolster the assertion that Usp11 modulates the survival of MZ B cells *via* Notch ligands.

In summary, Usp11 plays a pivotal role in modulating hematopoietic and immune system damage following ionizing radiation by influencing the ubiquitination of Notch’s ligands, DLL1 and JAG2, thereby promoting immune cell remodeling in the spleen (Graphical Abstract). We hypothesize that targeted inhibition of Usp11 may offer a promising strategy for intervention in the treatment or prevention of radiation-induced hematopoietic and immune systems damage. However, further in-depth mechanistic studies and extensive clinical trials with larger datasets are required to validate the clinical feasibility of Usp11 as a therapeutic target for hematopoietic and immune systems damage resulting from ionizing radiation.

## Materials and methods

### Mice

Female C57BL/6J (WT) mice were purchased from the Laboratory Animal Center of Shanghai (Shanghai, China). Usp11 knockout C57BL/6N mice (Usp11^−/−^) and their wild-type (WT) counterparts (Usp11^+/+^) were generated by Gem Pharmatech Biological Technology Co, Ltd. Mouse genotype was verified through both polymerase chain reaction (PCR) and Western blotting analyses at the mRNA and protein expression levels, as previously described [35]. All mice were maintained in SPF room at the animal facilities of Soochow University. During the experiment, all mice were randomized into groups using a random number table. All animal experimentation was adopting a single blind method and carried out following NIH guidelines under protocols approved by the Institutional Animal Care and Use Committee of Soochow University.

### Construction and assessment of irradiated mouse

6–8 weeks Usp11^−/−^ female mice and wild-type female mice were selected. The German Siemens KD-2 6MV X-ray linear accelerator was used for full body irradiation, with a total irradiation dose of 7.5 Gy and a dose rate of 200 cGy/min [18].

### Clinical scoring of radiation injury

The clinical scoring system for radiation-induced diseases encompasses a comprehensive array of parameters, including external characteristics, posture, mobility/vitality, appetite, dehydration, body weight, and body temperature. The evaluation criteria for each parameter are detailed as follows:

A. The condition of the hair. Scores range from 0 (normal hair) to 3 (very coarse hair), with intermediate scores of 1 for absent hair and 2 for coarse hair.

B. Signs of hunchback. A score of 0 indicates normal posture, while scores of 1, 4, and 6 correspond to mild hunchback, hunched head against the floor or prone in cage, and inability to maintain an upright posture, respectively.

C. Breathing and movement. A score of 0 indicates normal behavior and vitality, with scores of 1, 3, and 6 representing slightly reduced activity with subtle changes, changes in respiratory rate or vitality, and movement only when stimulated, respectively.

D. Appetite: Appetite is classified into four categories: normal, decreased appetite, not eating since the last check, and not eating at the last two checkpoints (assuming multiple daily checks). Scores range from 0 for normal appetite to 3 for not eating at the last two checkpoints.

E. Dehydration: Dehydration status is assessed as normal, mild, moderate, or severe. Scores of 0, 1, 2, and 3 are assigned, respectively, with mild and moderate dehydration requiring subcutaneous fluid and hydrogel administration.

F. Body Weight: Weight is monitored weekly initially, then every other day once a 10% change is observed, and daily after a 15% change. Scores range from 0 for an initial 5% weight change to 6 for a weight change exceeding 25%, with intermediate scores reflecting incremental weight loss.

G. Body Temperature: Body temperature is measured using an infrared thermometer. Scores of 0, 2, 4, and 6 are assigned for temperatures of 33–35 °C, 30–32.9 °C, 28–29.9 °C, and below 28 °C, respectively.

Euthanasia is considered when any single parameter reaches a score of 6 or when the composite score for parameters A to G exceeds or equals 15. Additional criteria for euthanasia include loss of consciousness, inability to maintain an upright posture, heavy breathing (gasping), and convulsions.

### Spleen resection

The skin and peritoneum were cut with sterilized ophthalmic scissors, the splenic artery of the mouse was found with ophthalmic forceps for ligation, and then the entire spleen was removed with ophthalmic scissors, and the peritoneum and skin were sutured.

### Single-cell sequencing

Mice spleen was dissociated for digestion, sieve filtration, and remove dead cells to obtain a single cell suspension. Adjust the concentration of the freshly prepared single-cell suspension to 700–1200 cell/μL and perform the operation and library construction according to the instructions of 10× Genomics Chromium Next GEM Single Cell 3ʹ Reagent Kits v3.1 (10x Genomics, USA). The constructed library is then subject to high-throughput sequencing using the Illumina Nova 6000 PE150 platform (OE Biotech, China) [36, 37].

### Single-cell RNA-seq data preprocessing

The bioinformatics analysis was provided by OE Biotech Co., Ltd. (Shanghai, China). Cell Ranger software (version 5.0.0) was used to process 10x Genomics genomic data and Unique Molecular Identifier (UMI) counting matrices using the R package Seurat [36] (version 3.1.1).

QC standards were applied to filter out cells with a gene number less than 200, a UMI less than 1000, and a log_10_GenesPerUMI less than 0.7. Subsequently, the DoubletFinder package (version 2.0.2) [37] was used to identify potential doublets. Cells were included in the downstream analysis and library size normalization was performed using the Normalize Data function in Seurat to obtain normalized counts [36].

The method described by Macosko et al. [38] was used to identify the top variant genes in a single cell. Most of the variable genes were selected using the Find Variable Genes function in Seurat’s article [36], principal component analysis (PCA) was performed using the RunPCA function to reduce the dimension. Use the Find Clusters feature to perform graph-based clustering of cells based on their gene expression profiles and visualize cells using the two-dimensional uniform manifold approximation and projection (UMAP) algorithm and the Run UMAP function. Positive markers are identified by identifying the marker genes for each cluster by the labeling function [36].

Subsequently, the cell origin of each single cell was inferred independently using R-package Single R (version 1.4.1) [39], referring to the transcriptome dataset “Mouse Primary Cell Atlas” to independently infer the origin cell of each single cell and identify the cell type [40–42]. Utilizing the Slingshot implemented in the R package, we constructed the single cell pseudo-time trajectory for each sample [43]. The methodology of Slingshot involves a cluster-based minimum spanning tree approach for lineage inference and utilized the simultaneous principal curves method to infer pseudotime [44]. Data of single cell sequencing had been deposited at https://ngdc.cncb.ac.cn/bioproject/browse/PRJCA026939.

### Histopathology and immunohistochemistry

Mouse spleen and femur were obtained at certain time points. After fixed in 4% paraformaldehyde and embedded in paraffin, tissues were cut in section (5μm in thickness), dehydration, transparency, immersion and embedding, sectioning, patching, dewaxing and hydration, stained with hematoxylin and eosin (H&E), section dehydration and examined under a light microscope [18]. Semi-quantitative analysis was performed using image J software. The ratio of MZ was calculated by dividing the width of the marginal zone by the distance from the outermost end of the marginal zone to the center point of the white pulp. Tissue damage was assessed based on a pathological scoring system (Histology score) [45–49]. For immunohistochemical analysis, spleen sections were immunostained with antibodies specific to CD3, CD169, and CD20 (Servicebio, China) (Supplementary Table S1). The immunohistochemical cumulative optical density (IOD), a metric that aggregates the optical density values of individual brown staining spots within the image, was employed to quantitatively assess the expression level of the targeted proteins through optical densitometry. The presence of brownish-yellow particles indicated positive cellular staining. The IOD measurements were precisely obtained using ImageJ software.

### Immunofluorescence

Mouse spleen was collected at specific time intervals and prepared for immunohistochemical examination. Tissue was embedded with OTC and sliced with a frozen microtome (5 μm). Spleen sections are stained overnight at 4 °C with α-SMA (1:100 Biorigin, China) with CD3(1:500 Servicebio, China) or CD169(1:500 Servicebio, China) or CD20 antibodies (1:3000 Servicebio, China) (Supplementary Table S1), after PBS washing, perform room temperature staining with fluorescent secondary antibodies of different colors (α-SMA: Cy3, red; CD3, CD169 and CD20: FITC, green). (Servicebio, China). Imaging was conducted using a confocal microscope. The mean fluorescence intensity (MFI), a quantitative metric representing the average fluorescence signal emitted by a fluorochrome on the cellular or particulate surface, was calculated.

## ELISA

Serum and tissue cell suspensions of the recipient mice at certain time points were obtained and stored at −80 °C. Serum IgM and IgG were validated by mouse IgM ELISA assay (Solarbio, China) and IgG ELISA assay (Neobioscience, China) according to manufacturer’s protocols [50].

### Flow cytometry

Antibodies used for flow cytometry staining include anti-CD45-Alexa Fluor 700 (1:400 BioLegend, USA), anti-CD19-APC (1:400 BioLegend, USA), anti-CD23-PE-Cy7 (1:400 eBioscience, USA), anti-CD21-FITC (1:400 BioLegend, USA), and anti-CD45R(B220) (1:400 BioLegend, USA), anti-IgM-FITC (1: 400 eBioscience, USA) (Supplementary Table S1). All the results were analyzed by Flowjo software. Mouse spleen was acquired at a specific time point, then ground and filtered, and the single-cell suspension was collected and split red and stained after counting. Add the corresponding antibody, mix well, incubate on ice for 20 min in the dark, and wash and resuspend with PBS [51]. B cells were sorted by CD45-Aleax Fluor, B1 and B2 cells were separated by CD19-APC and B220-Cy5.5, and MZ B cells and FO B cells were separated by CD21-FITC and CD23-PE in B2 cells for detection and analysis.

### Cell culture and transfection

HEK-293T cells were cultured in DMEM medium with 10% fetal bovine serum (FBS) and 1% anti-penicillin and streptomycin at 37 °C in a humidified atmosphere with 5% CO_2_. The plasmid was transfected with Lipo3000 transfection reagent (Invitrogen, China). Usp11 overexpression plasmids (Ad-Usp11-Flag) and control plasmids were packaged using the adenovirus vectors (WZ Biosciences, China). DLL1(DLL1-Myc), JAG2(JAG2-Myc) and ubiquitin (Ub-HA) over-expression plasmids were purchased from Genechem (Genechem, China).

### Co-immunoprecipitation

The extracted fresh protein samples are added to the antibody according to the volume ratio and incubated overnight at 4 °C by spinning; after protein A/G beads (MCE, USA) were added, samples were incubated in room temperature for 4 h; washed beads with PBST, discard supernatant, denature beads plus SDS with RIPA-lysate, immediately perform Western Blot detection. Antibodies targeting Flag, Myc was purchased from Proteintech (Proteintech, USA), antibodies targeting Ub from Abcam (Abcam, USA), Mouse IgG from Biyuntian (Biyuntian, China) (Supplementary Table S1)

### Database analysis

The GDS2762 database was analyzed to compare the expression of Usp11 in B cells stimulated by IFN-β and in B cells that were not stimulated, and the GDS2554 database was analyzed to compare the expression of Usp11 in normal resting B cells and aggressive lymphoma sample B cells. The potential correlation between Usp11 and Notch ligands (DLL1 and JAG2) was analyzed in the TCGA and GTEx database.

### Statistical analysis

All data are presented as means ± S.D. Statistical analysis between two groups was done using Student’s *t* test with the GraphPad Prism software. Statistical significance was determined by *p* values < 0.05.

## Supplementary information

supplement file

original data

## Data Availability

All data within the article and its supplementary information files that support this study are available from the authors upon request. Data of single cell sequencing had been deposited at https://ngdc.cncb.ac.cn/bioproject/browse/PRJCA026939.
