# Supplementary material for: Usp11 maintained the survival of marginal zone B cells under ionizing radiation by deubiquitinating DLL1 and JAG2
Source: Cell Death Dis. 2025 Feb 4;16(1):67. doi: 10.1038/s41419-025-07377-7 (PMC11794699; doi:10.1038/s41419-025-07377-7)
Supplement: Supplementary file 1 — supplement file [file 41419_2025_7377_MOESM1_ESM.pdf]

**Supplement Figure legend**

**Supplement Fig. S1. Change of body weight and clinical score of mice after total body irradiation. A.** Trend of body weight of Usp11$^{+/+}$ and Usp11$^{-/-}$ mice after 7.5 Gy irradiation. **B.** Clinical score of mice after total body irradiation**.** Mice were subjected to systemic 7.5 Gy irradiation, were clinically scored, and a linear fit was performed. Note: The irradiation dose rate is 2.00 Gy/min.

**Supplement Fig. S2. Bone marrow change in mice after irradiation. A.** Bone marrow pathology in mice after irradiation. On the 0th, 6th, and 12th days after 7.5 Gy TBI, the femurs of mice were taken and fixed in 4% paraformaldehyde solution, followed by decalcification, followed by paraffin-embedded sections, HE staining, and the pathological changes of the tissues were observed under the microscope (n=3). Note: Scale bars 500 μm and 100 μm; The irradiation dose rate was 2.00 Gy/min. **B.** Bone marrow pathological score of mice after irradiation. The pathological changes of bone marrow HE staining on days 0, 6 and 12 after 7.5 Gy TBI in mice were counted.

**Supplement Fig. S3. Distribution of macrophages in the spleen. A.** Immunohistochemistry was used to compare the changes of CD169$^{+}$ macrophages in the spleen of Usp11$^{+/+}$ and Usp11$^{-/-}$ mice on days 0, 6, and 12 after TBI. Scale bars are 500 μm, 200 μm. **B.** Statistical analysis of CD169 immunohistochemical IOD values in mouse spleen. **C.** Immunofluorescence was used to compare the changes of macrophages in the marginal region of the spleen of Usp11$^{+/+}$ and Usp11$^{-/-}$ mice on days 0 and 6 after TBI. Scale bar is 500 μm. **D.** Statistical analysis of the proportion of CD169 immunofluorescence in mouse spleen.

**Supplement Fig. S4. Distribution of T cells in the spleen. A.** Immunohistochemistry was used to compare the changes of CD3$^{+}$ T cells in the spleen of Usp11$^{+/+}$ and Usp11$^{-/-}$ mice on days 0, 6, and 12 after TBI. Scale bars are 500 μm, 200 μm. **B.** Statistical analysis of CD3 immunohistochemical IOD values in mouse spleen. **C.** Immunofluorescence was used to compare the changes of T cells in the spleen of

Usp11$^{+/+}$ and Usp11$^{-/-}$ mice on days 0 and 6 after TBI. Scale bar is 500 μm. **D.** Statistical analysis of the proportion of CD3 immunofluorescence in mouse spleen.

**Supplement Fig. S5. Usp11 in regulating B lymphocyte-associated biological processes. A.** The expression of Usp11 in IFN-β-activated B cells was compared by GDS2762 database analysis. **B.** The expression of Usp11 in B cells of aggressive lymphoma samples was compared by GDS2554 database analysis. *$p$<0.01, ***$p$<0.001.

**Supplement Fig. S6. Expression level of Usp11 in different types of B cells in single-cell sequencing data**

**Supplement Fig. S7. The MZ B cluster enriched in the Usp11$^{-/-}$ samples post-irradiation**. **A**. The expression of the marker genes with the most prominence in each subgroup. **B**. Pseudo-time trajectory analysis B cell groups of mice spleen.

**Supplement Fig. S8. Splenic T cells and macrophage cells between Usp11$^{-/-}$ and Usp11$^{+/+}$ mice on day 6 post-TBI.** Proportion of T cells and macrophage cells analyzed using flow cytometry.

**Supplement Fig. S9. γ H2AX expression levels in B cells in the spleen post-irradiation.** Splenic cells were cultured in vitro. After exposure to 1Gy TBI, γH2AX expression levels were detected by FCM at both 2 and 6 hours post-irradiation. FO B: follicular B cell; MZ B: marginal zone B cell.

**Supplement Fig. S10. IgM and IgG in serum of mice. A.** IgM in serum of Usp11$^{+/+}$ and Usp11$^{-/-}$ mice at 0, 6, and 12 days after 7.5 Gy TBI were compared using ELISA assays. **B.** IgG in serum of Usp11$^{+/+}$ and Usp11$^{-/-}$ mice at 0, 6, and 12 days after 7.5 Gy TBI were compared using ELISA assays.

**Supplement Fig. S11. Distribution of macrophages in the spleen after MIX intervention. A.** CD169$^+$ cells in the spleen of Usp11$^{+/+}$ versus Usp11$^{-/-}$ mice treated with or without MIX intervention for three days after TBI. **B.** Statistical analysis of immunofluorescence staining of spleen CD169$^+$ cells.

**Supplement Fig. S12. Distribution of T cells in the spleen after MIX intervention. A.** CD3$^+$ cells in the spleen of Usp11$^{+/+}$ versus Usp11$^{-/-}$ mice treated with or without MIX intervention for three days after TBI. **B.** Statistical analysis of immunofluorescence staining of spleen CD3$^+$ cells.

**Supplement Fig. S13.** IgM changes in serum of Usp11$^{+/+}$ and Usp11$^{-/-}$ mice treated with or without MIX intervention for three days after TBI on days 0 and 6 after TBI.

**Supplement Fig. S14. The expression of Usp11 correlated with DLL1 and JAG2**. **A.** Usp11 expression positively correlated with DLL1 expression in normal whole blood and spleen. **B.** Usp11 expression positively correlation with DLL1 in AML databases. **C.** Usp11 expression positively correlated with JAG2 expression in normal whole blood and spleen. In the AML database (**D**) and DLBC (diffuse large B-cell lymphoma) dataset (**E**) Usp11 expression related to JAG2.

**Supplement Fig. S15.** Interactions of Usp11 with DLL1 (**A**) and JAG2 (**B**) in splenic tissue of mice

**Supplement Fig. S16.** Quantitative data of Fig8B (**A**) and Fig8D (**B**).

**Supplement Table S1 Antibodies information**

| Name | Catalog numbers | Company |
|---|---|---|
| **Flow cytometry** | | |
| CD19-APC | 115512 | Biolegend |
| B220-Percp-Cy5.5 | 103235 | Biolegend |
| CD21-FITC | 115506 | Biolegend |
| CD23-PE-Cy7 | 25-0232-82 | eBioscience |
| CD45- Alexa Fluor 700 | 103132 | Biolegend |
| IgM-FITC | 11-5790-81 | eBioscience |
| **IF&IHC** | | |
| CD3 | GB12014 | Servicebio |
| CD169 | GB11299 | Servicebio |
| α -SMA | BN42131R | Biorigin |
| CD20 | GB11540 | Servicebio |
| **Co-IP** | | |
| Flag | 66008 | Proteintech |
| c-MYC | 10828 | Proteintech |
| Ubiquitin | ab134953 | Abcam |

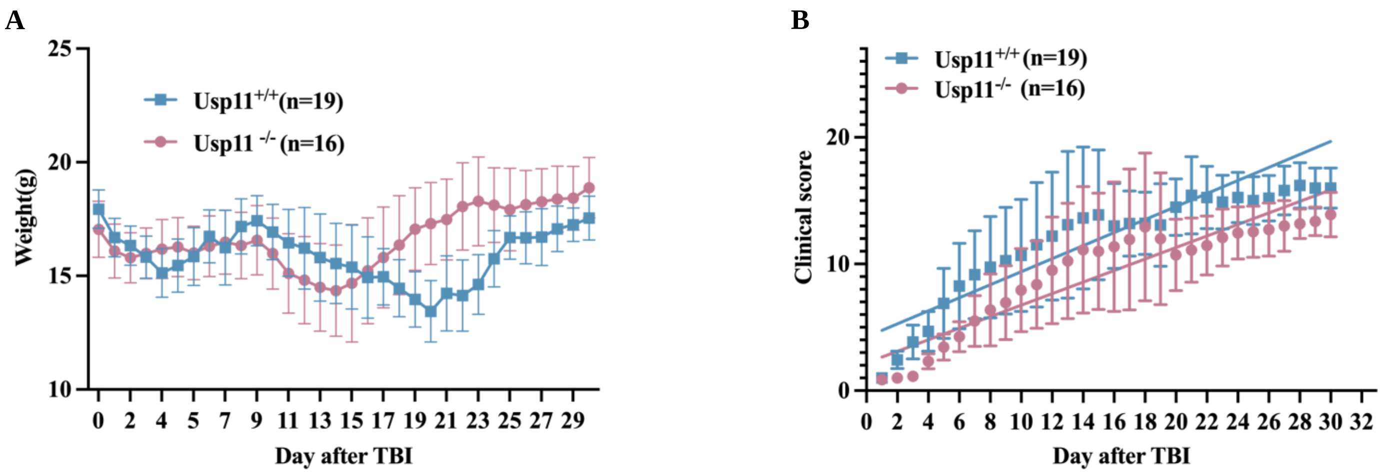


**Supplement FigS1**

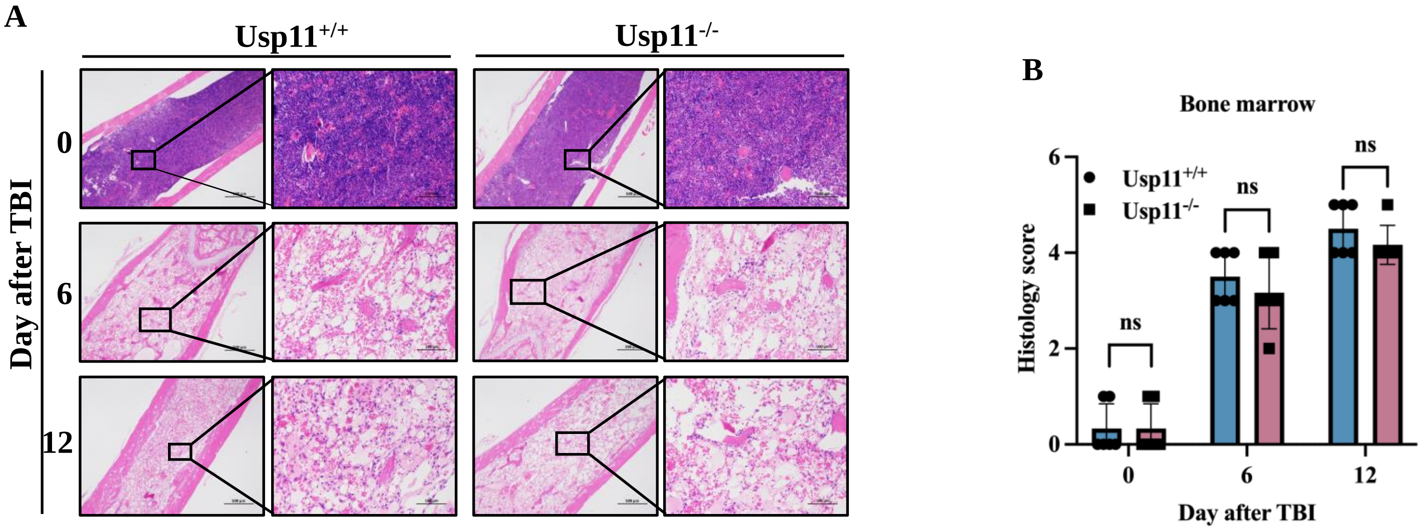


Supplement FigS2

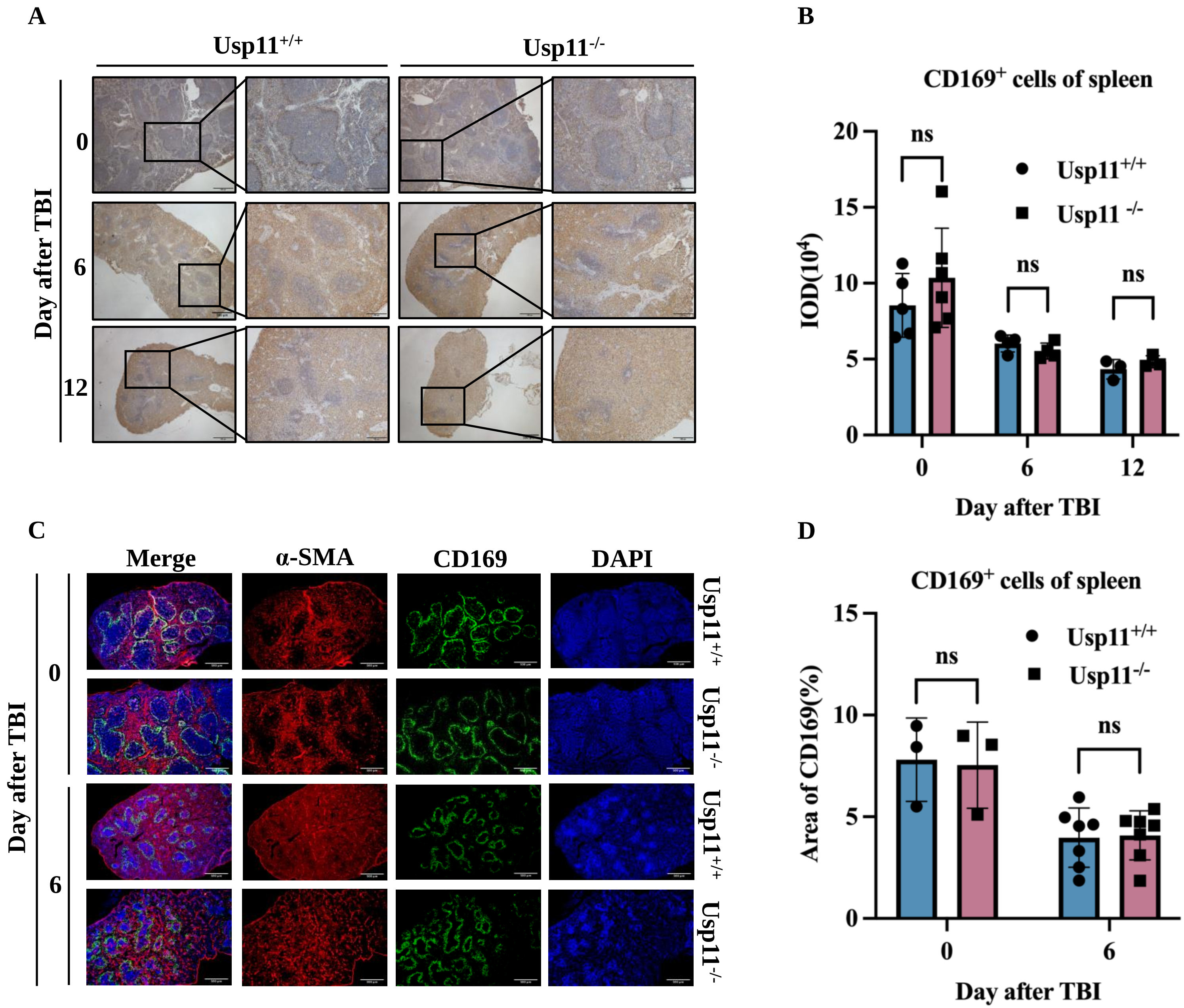


Supplement FigS3

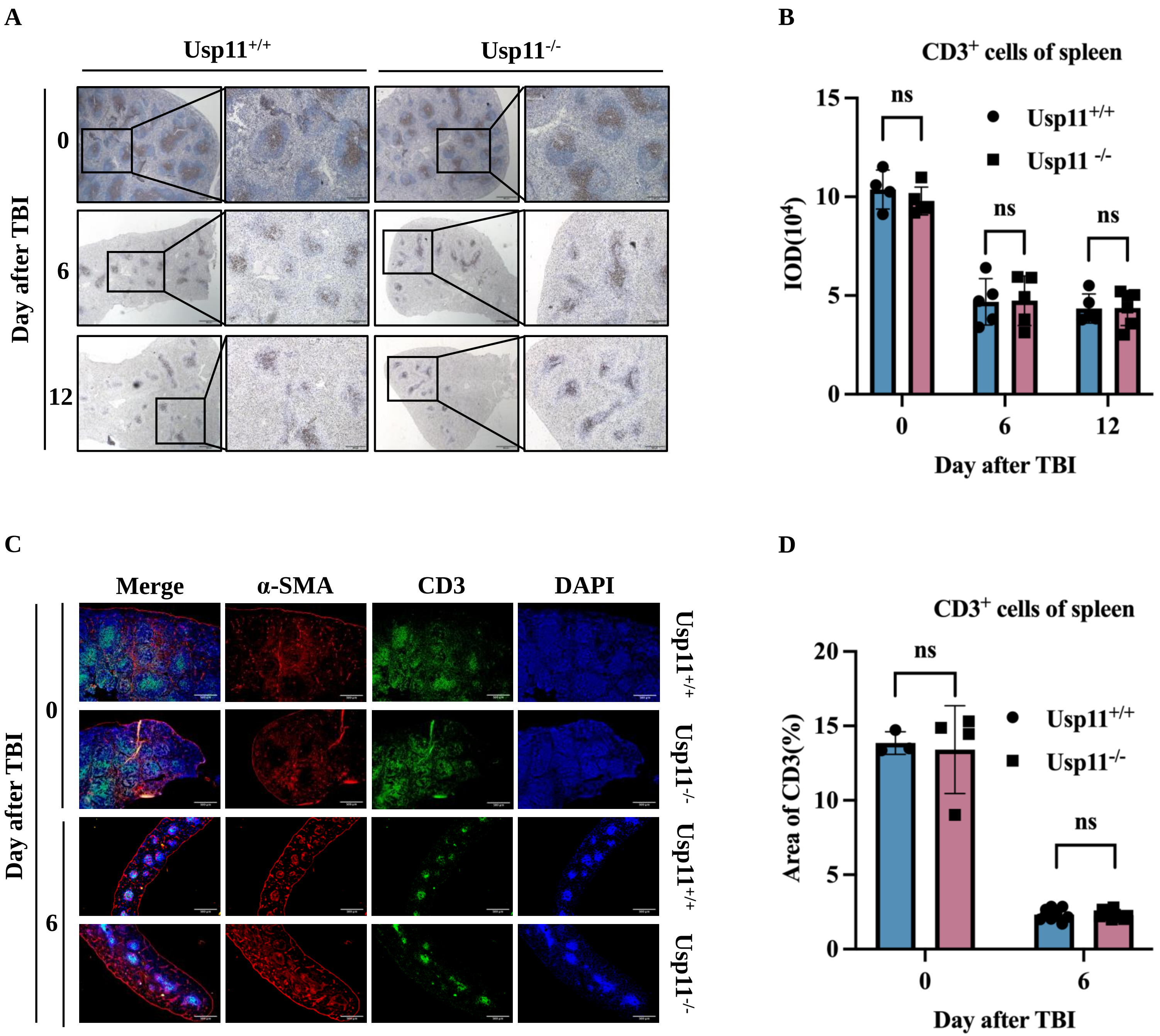


Supplement FigS4

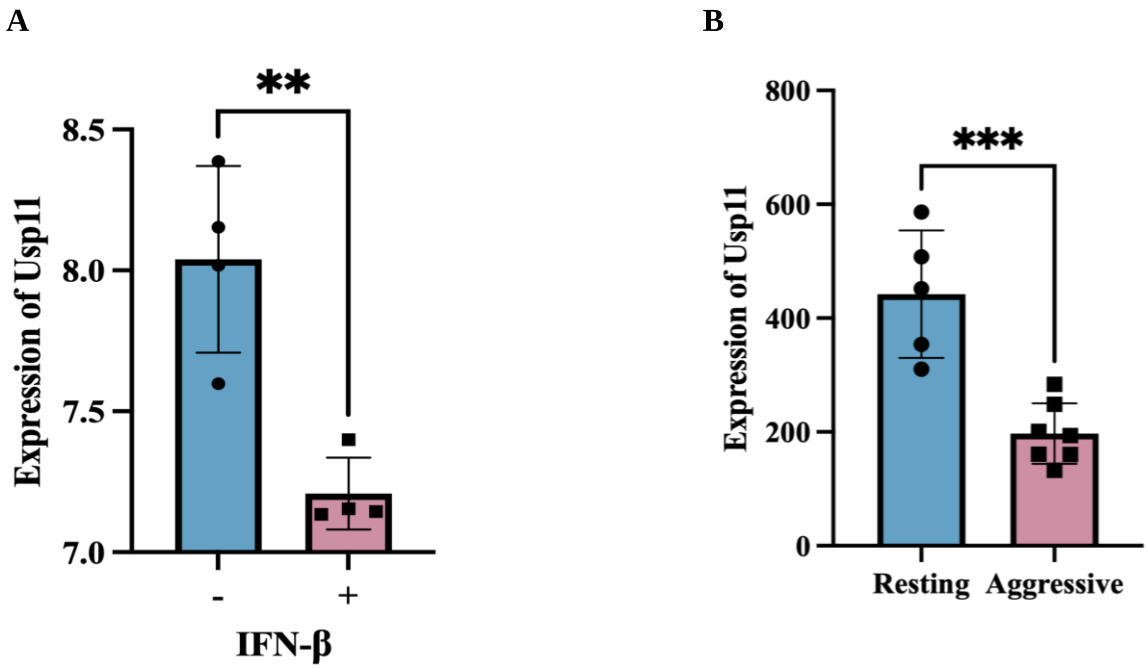


**Supplement FigS5**

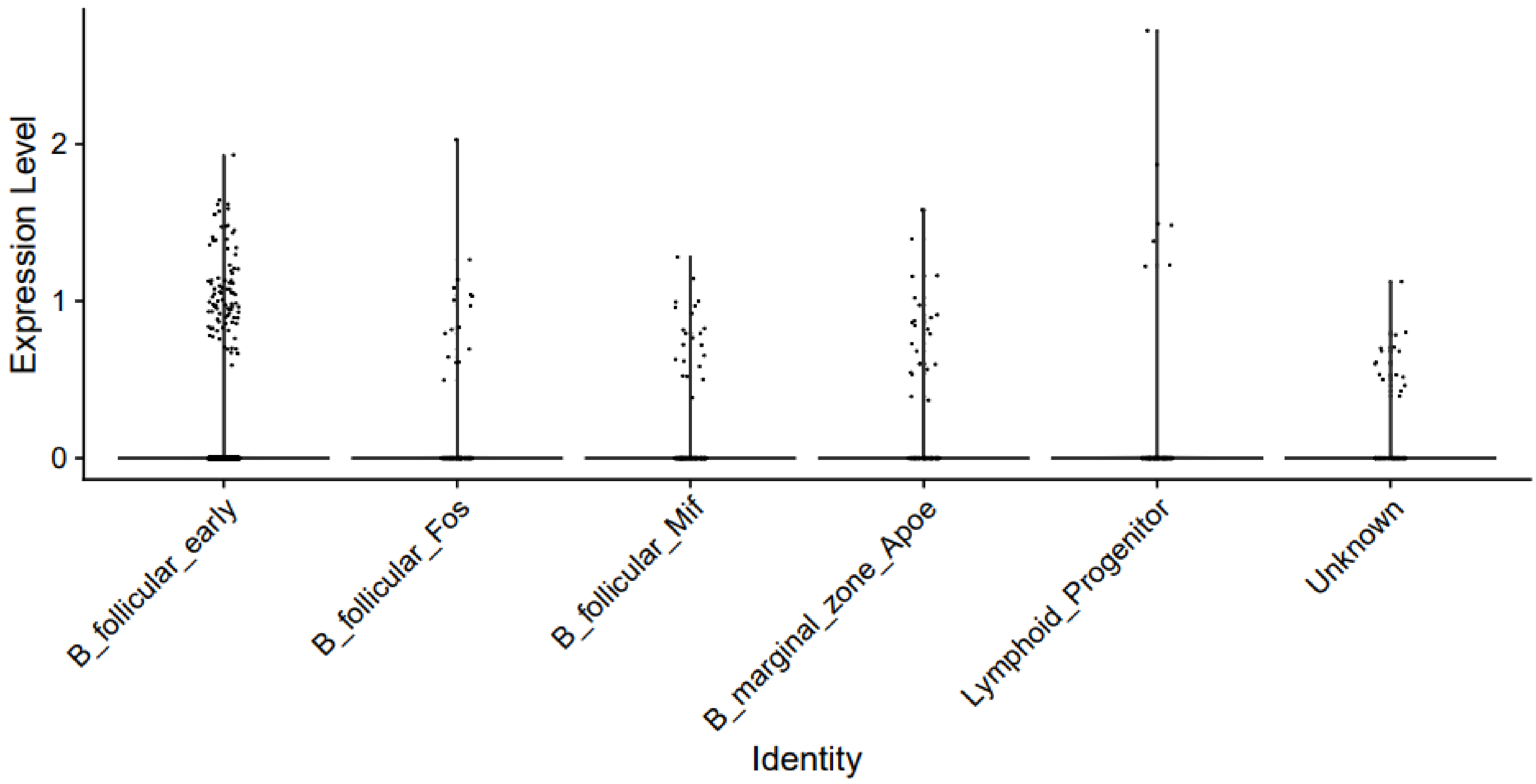


**Supplement FigS6**

**A**

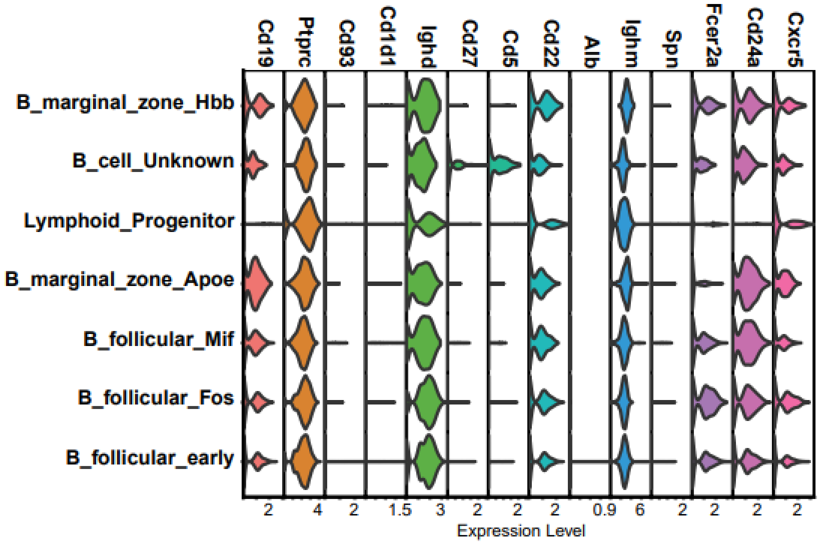


**B**

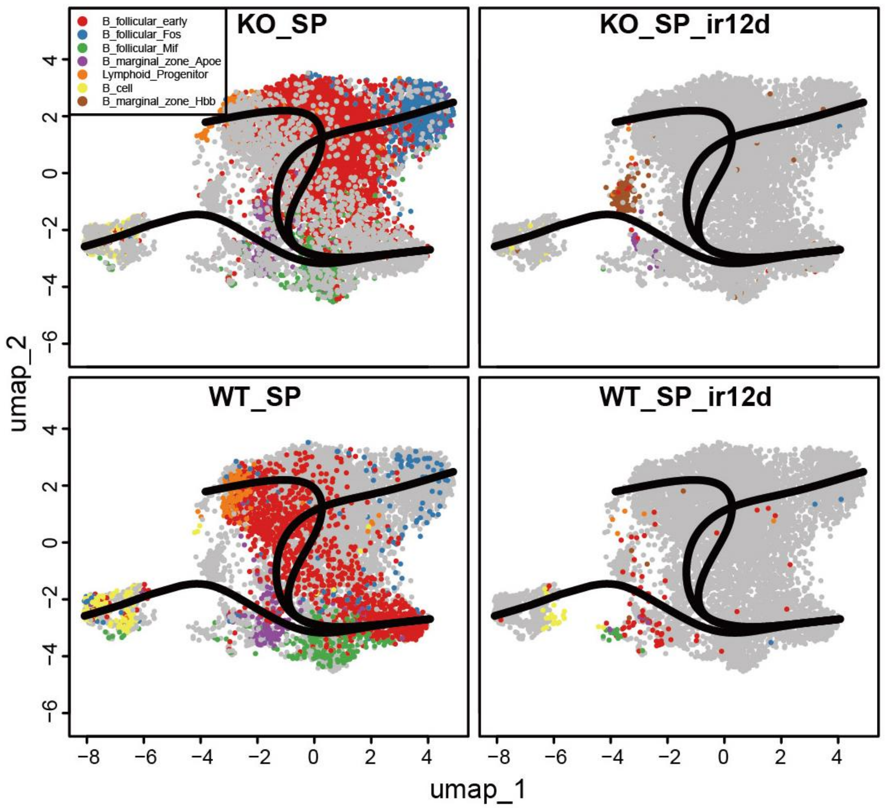


**Supplement FigS7**

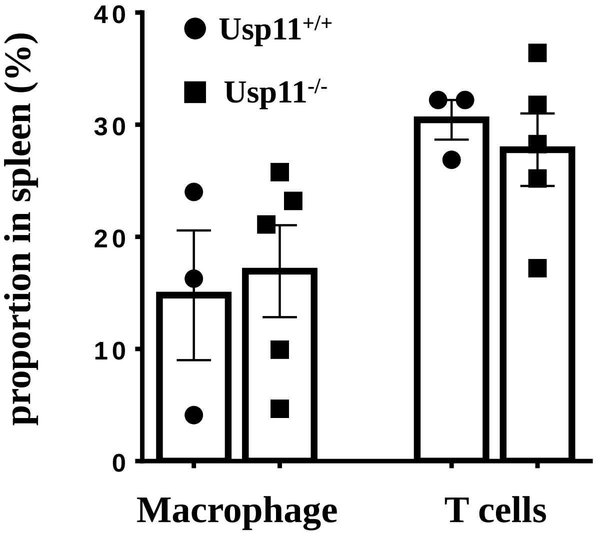


Supplement FigS8

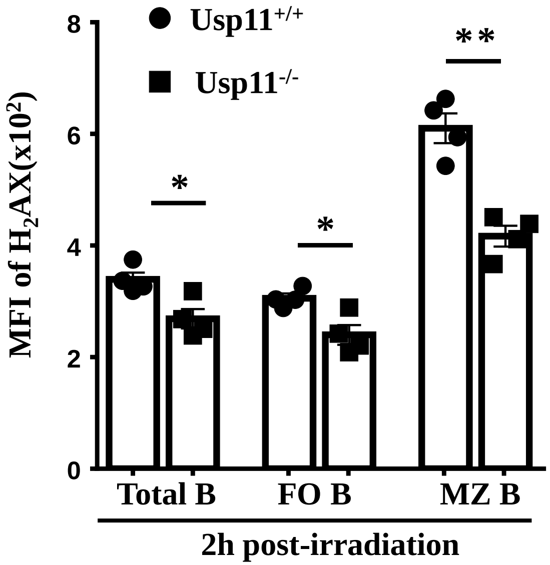


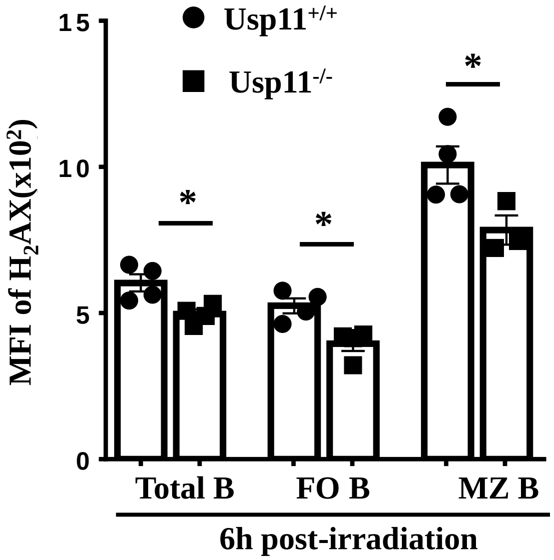


**Supplement FigS9**

A

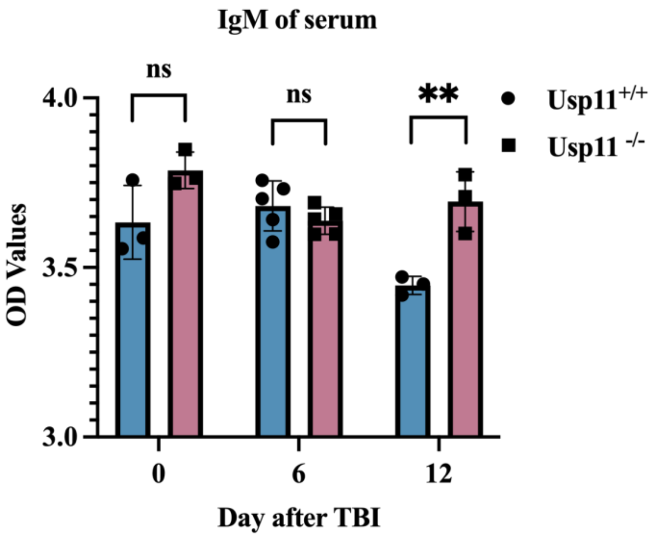


B

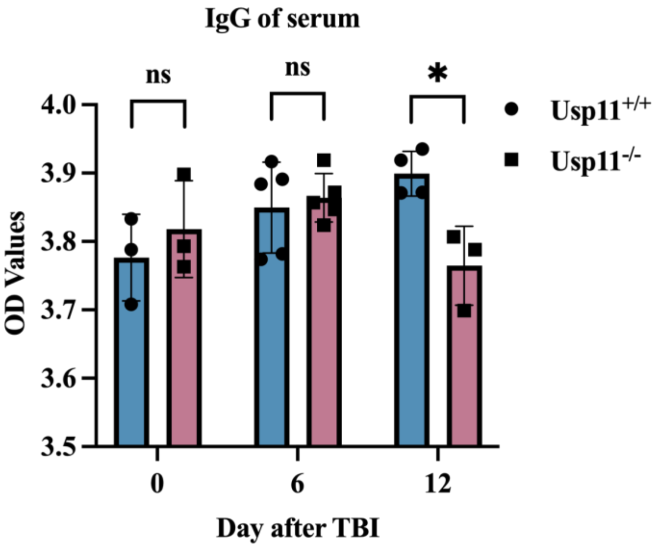


Supplement FigS10

A

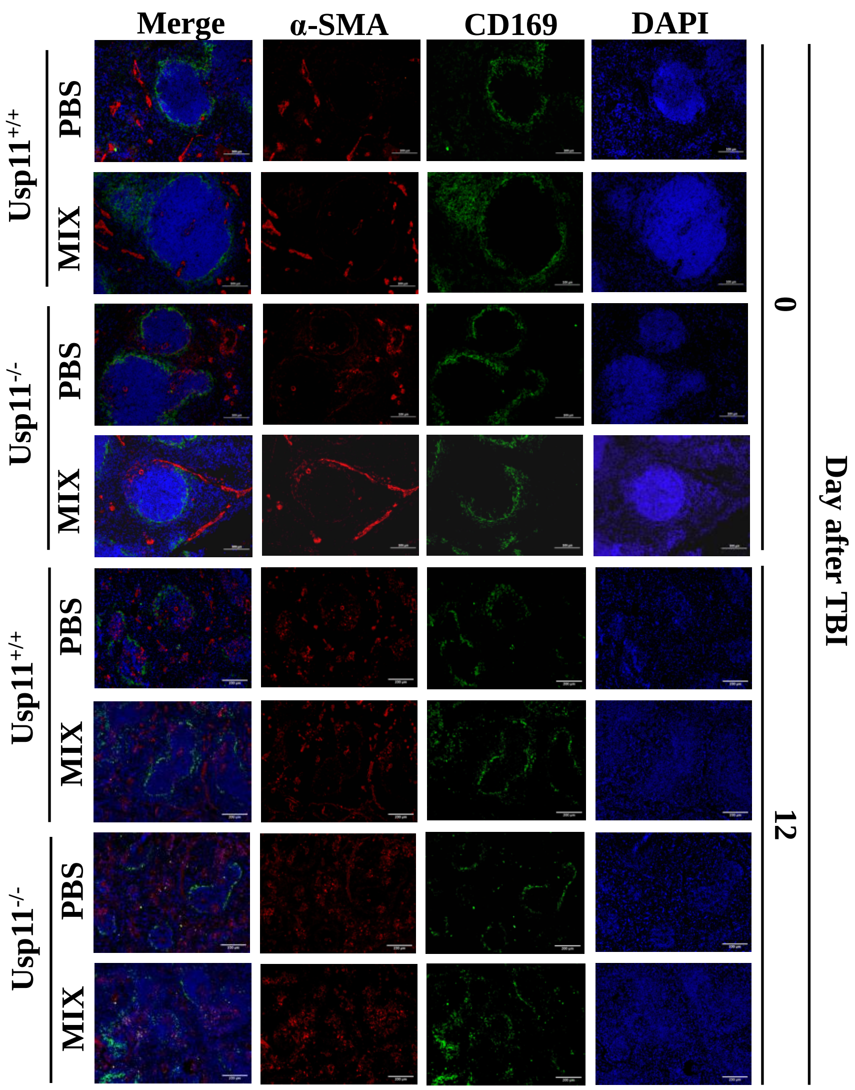


B

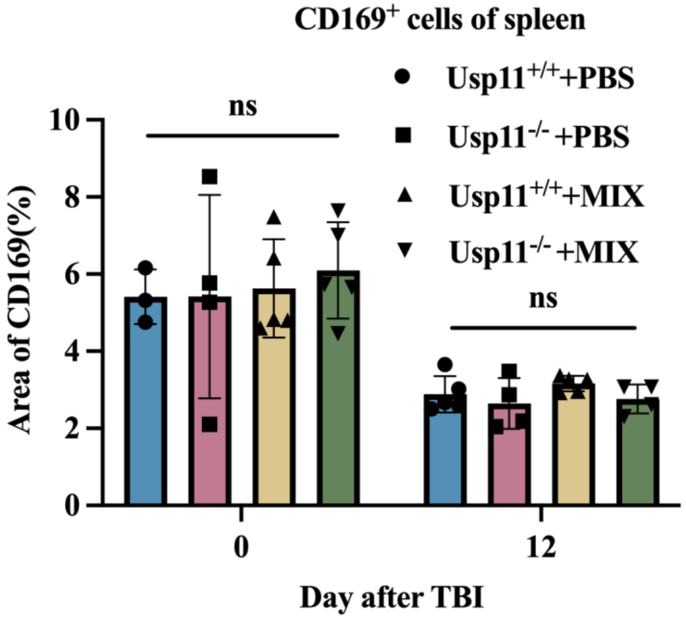


Supplement FigS11

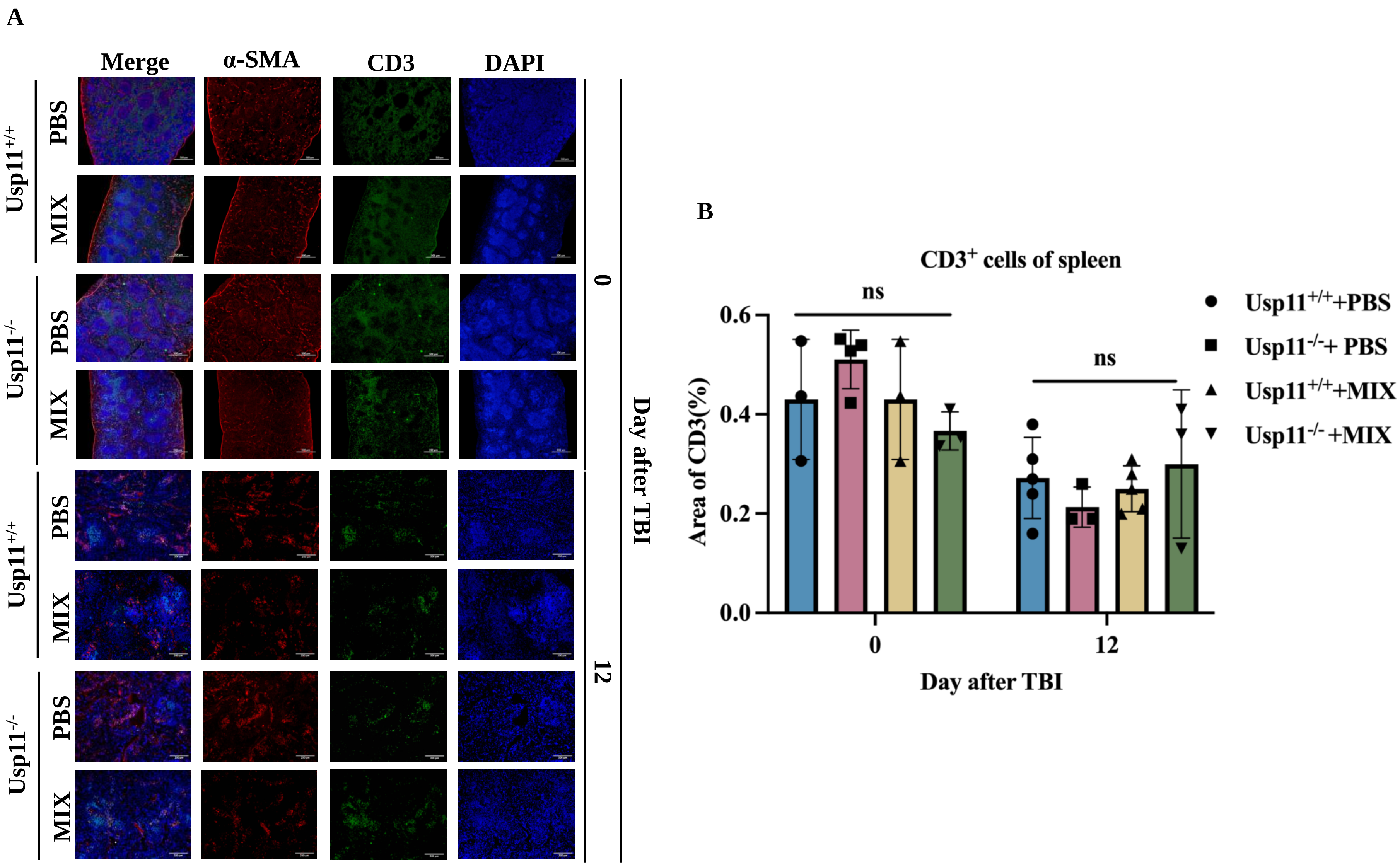


Supplement FigS12

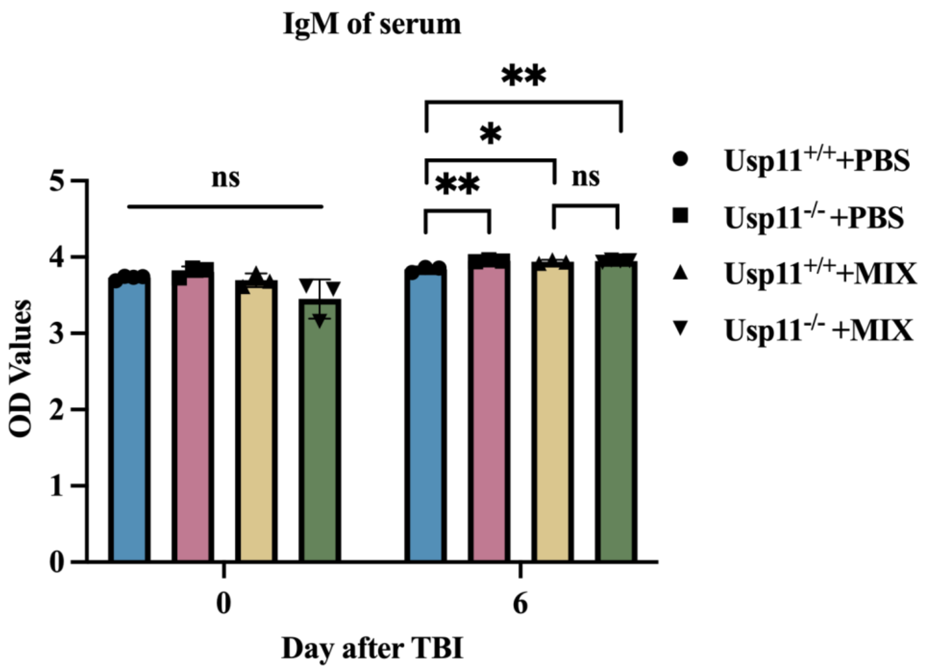


**Supplement FigS13**

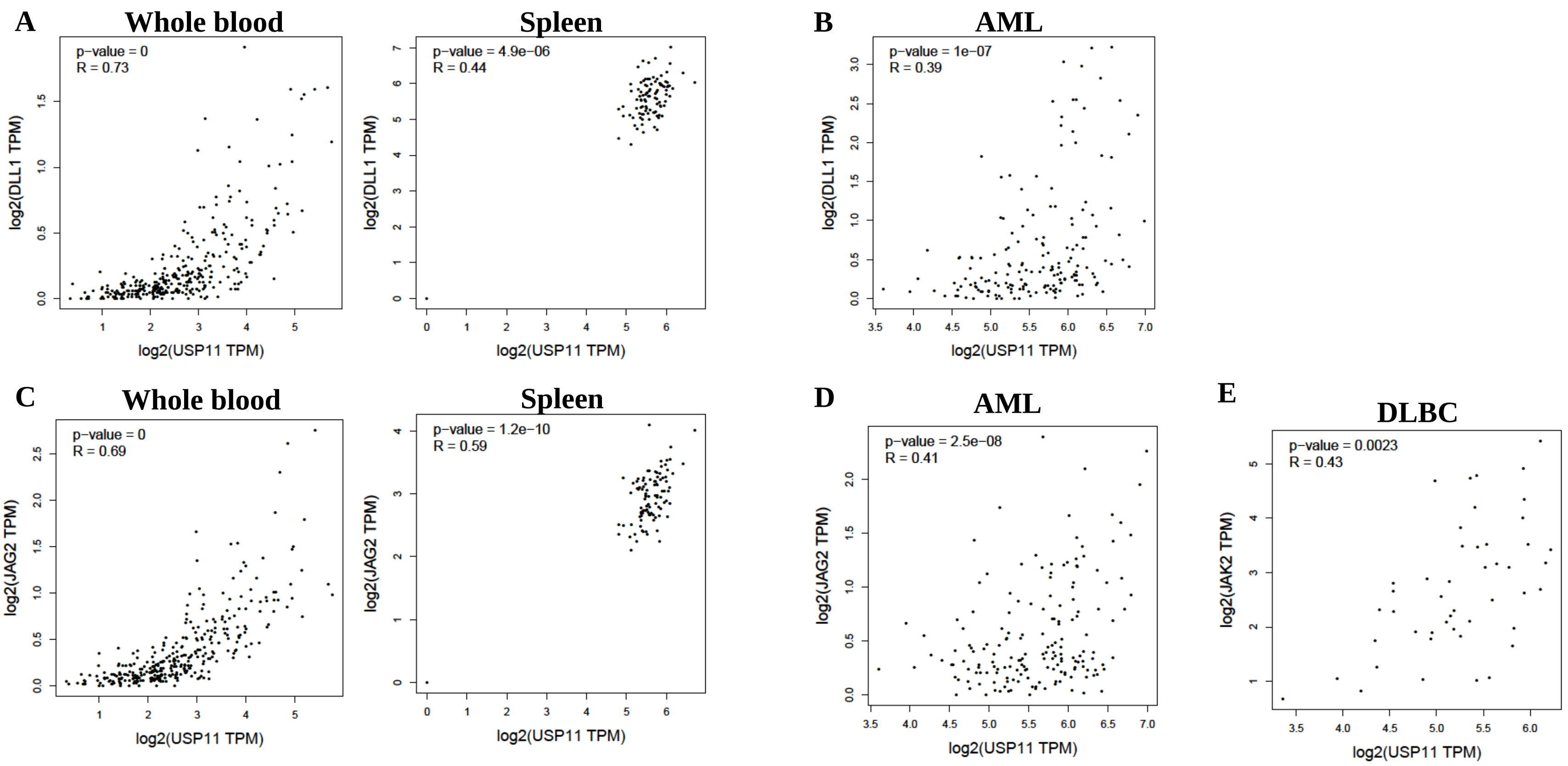


**Supplement FigS14**

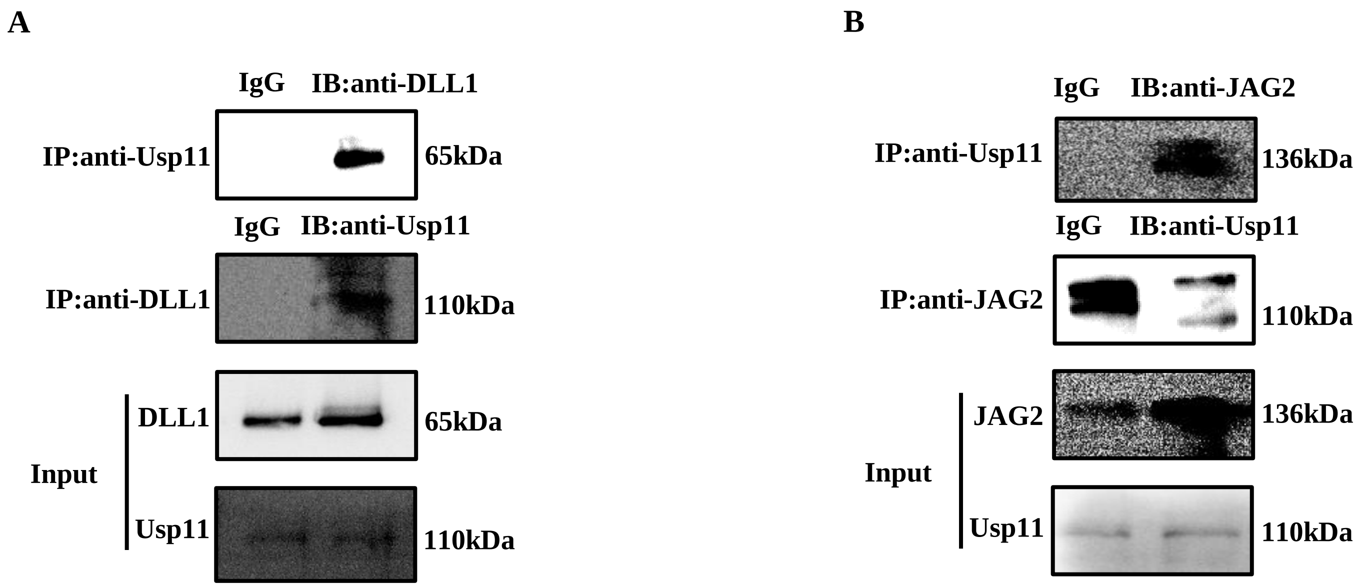


Supplement FigS15

A

B

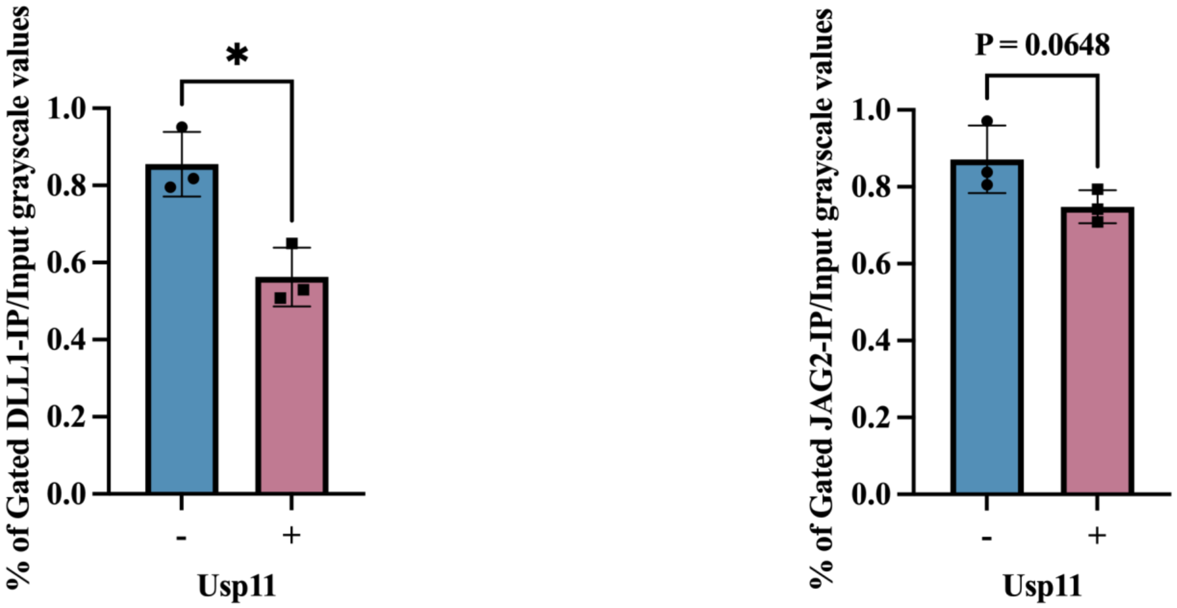


**Supplement FigS16**
