# Supplementary material for: Usp11 maintained the survival of marginal zone B cells under ionizing radiation by deubiquitinating DLL1 and JAG2
Source: Cell Death Dis. 2025 Feb 4;16(1):67. doi: 10.1038/s41419-025-07377-7 (PMC11794699; doi:10.1038/s41419-025-07377-7)
Supplement: Supplementary file 2 — original data [file 41419_2025_7377_MOESM2_ESM.pdf]

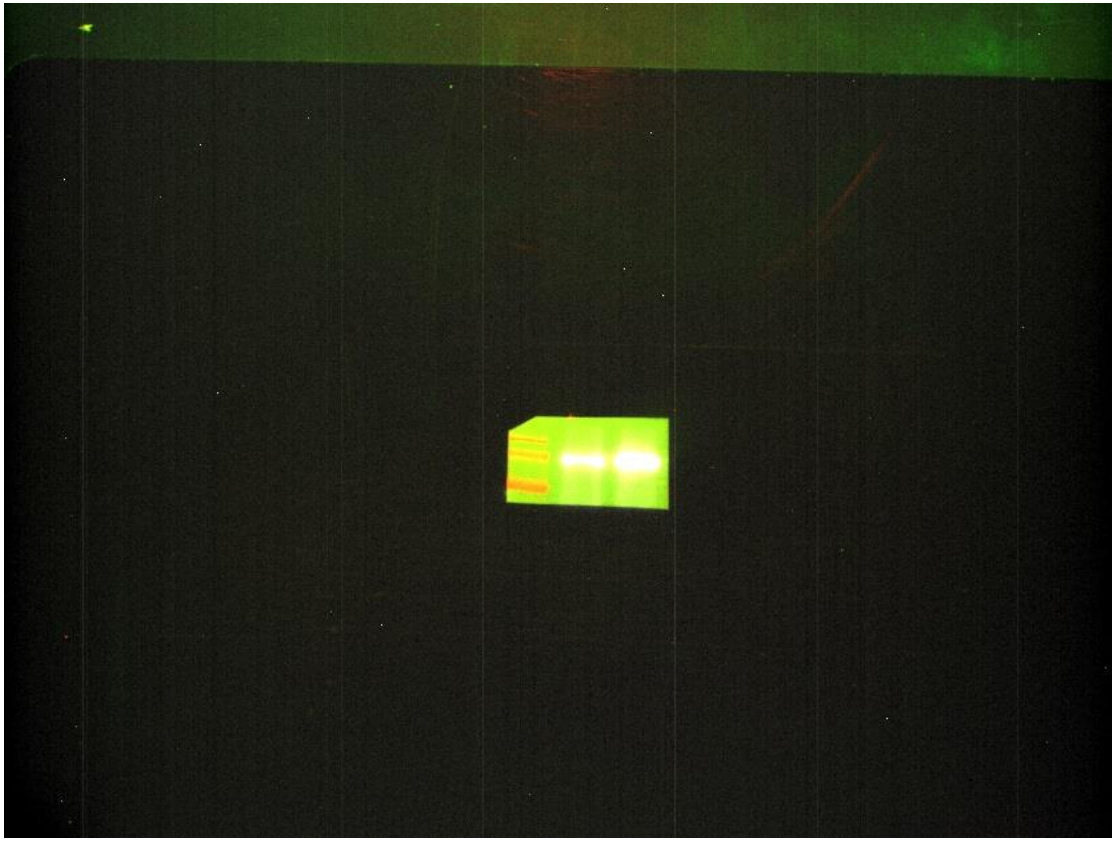

Fig 8A FLAG input

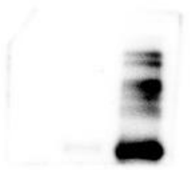

Fig 8A MYC IB FLAG IP

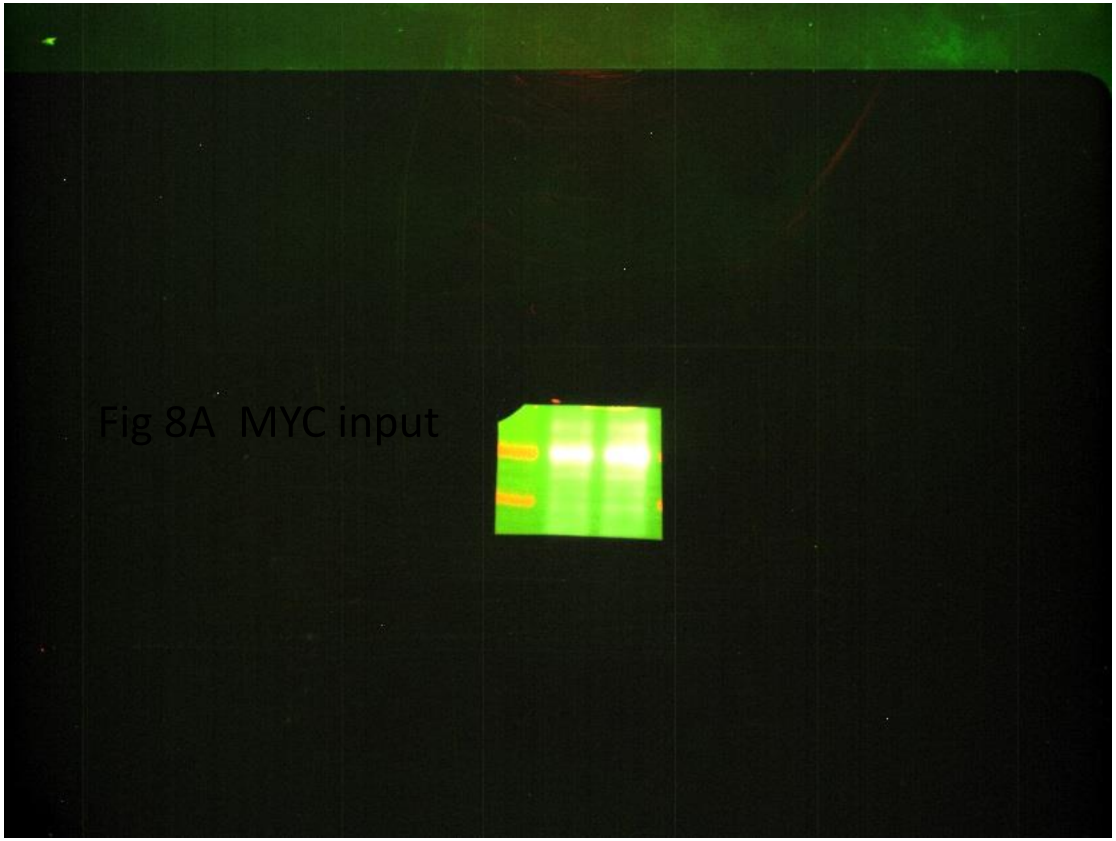

Fig 8A MYC input

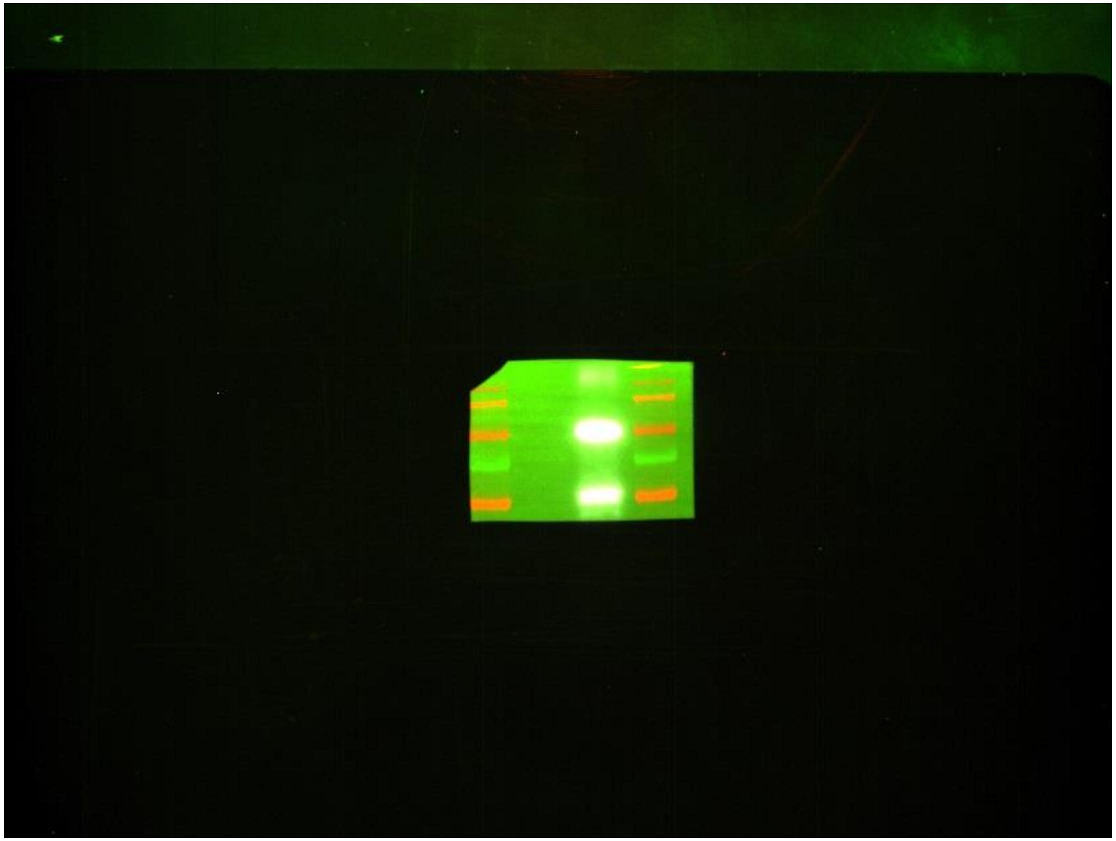

Fig 8A MYC IP FLAG IB

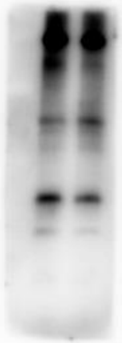

Fig 8B MYC IP UB WB

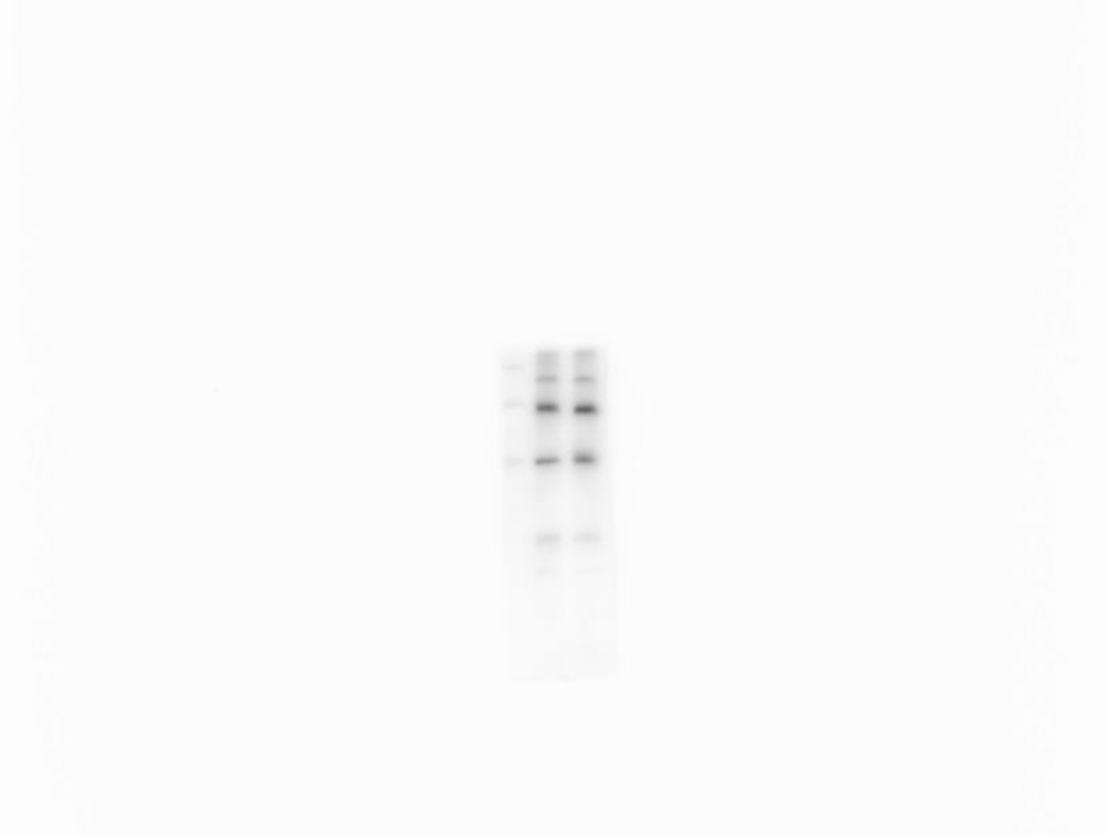

Fig 8B MYC WB

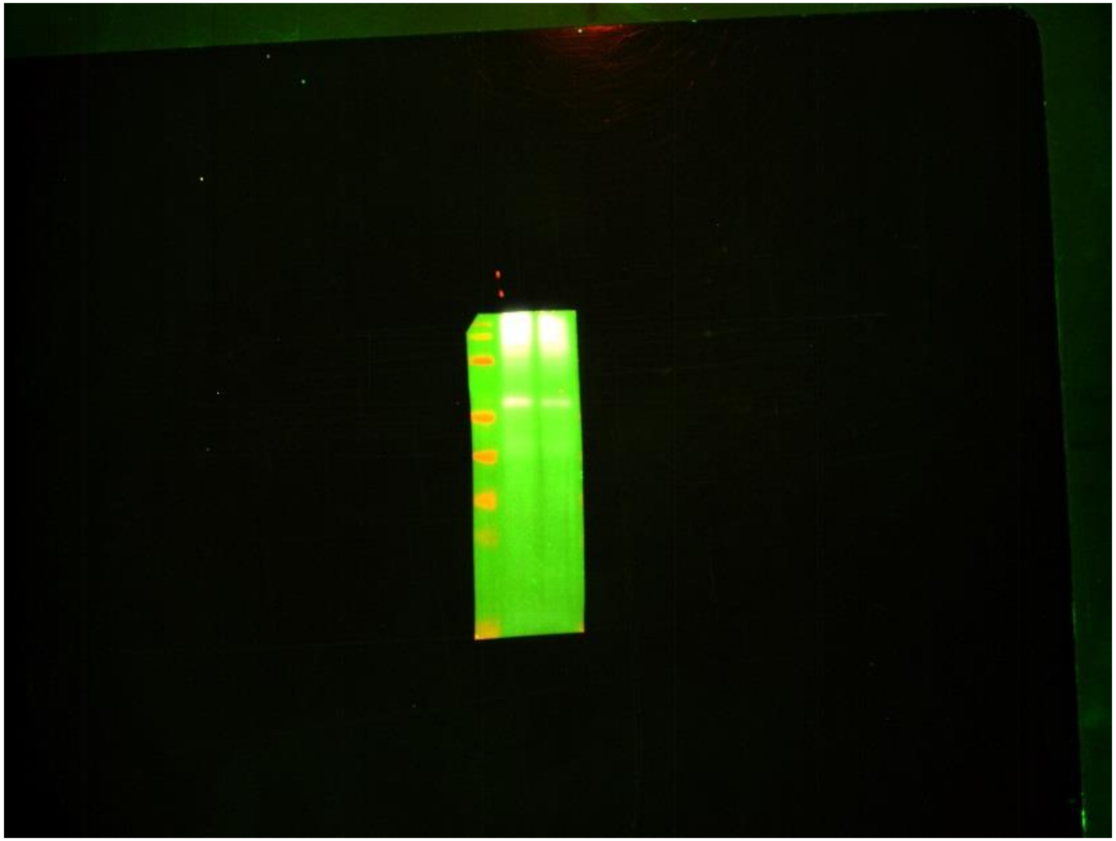

Fig 8B UB input

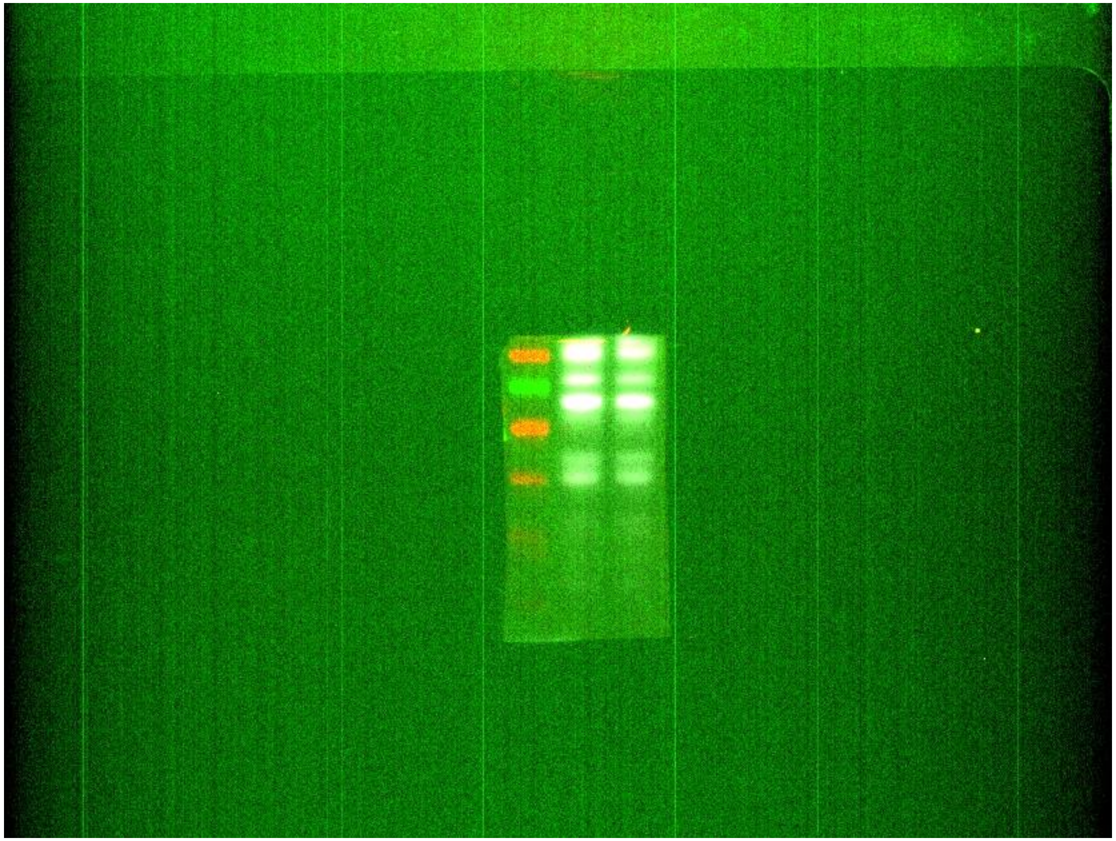

Fig 8C FLAG input

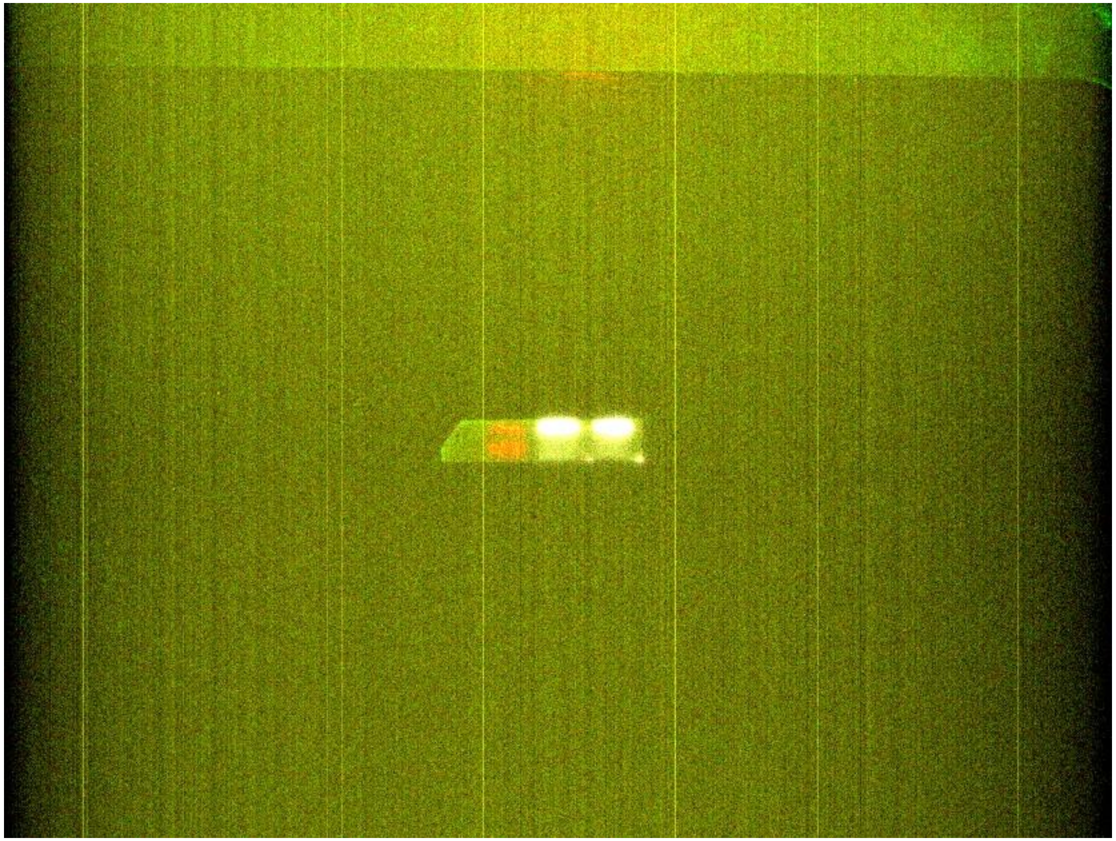

Fig 8C MYC input

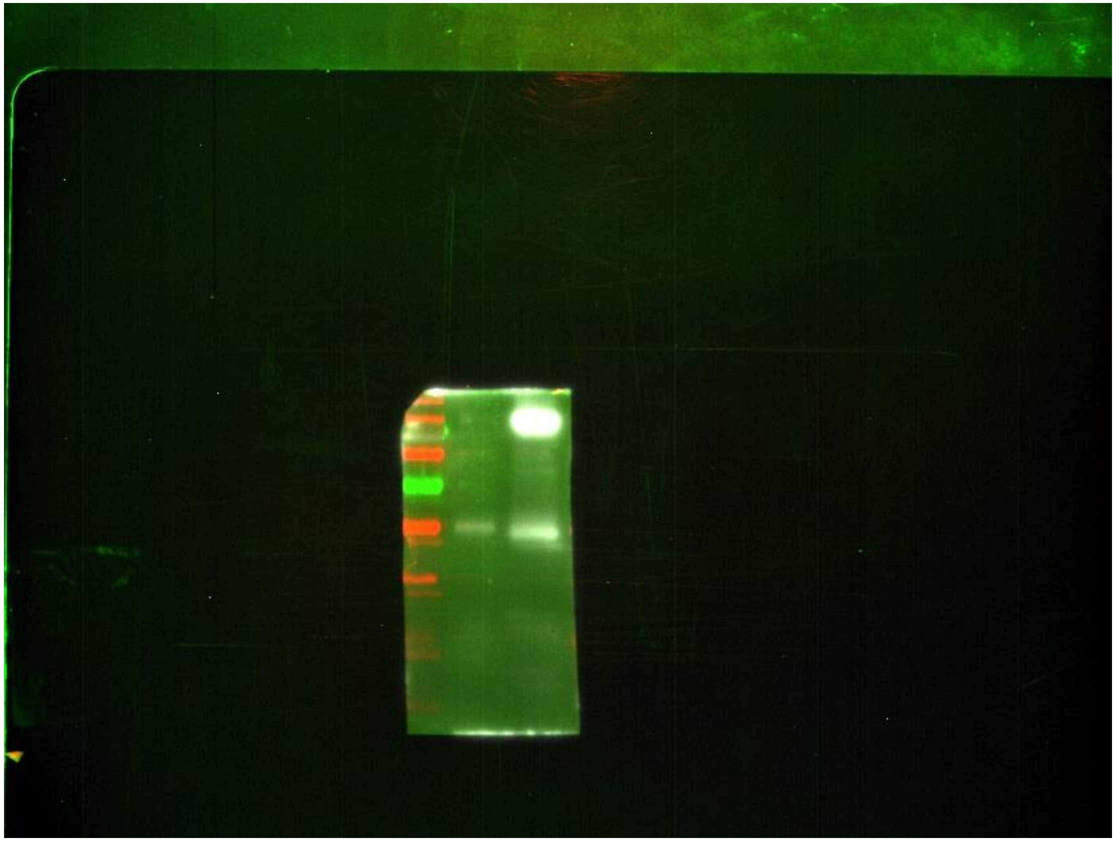

Fig 8C MYC IB FLAG IP

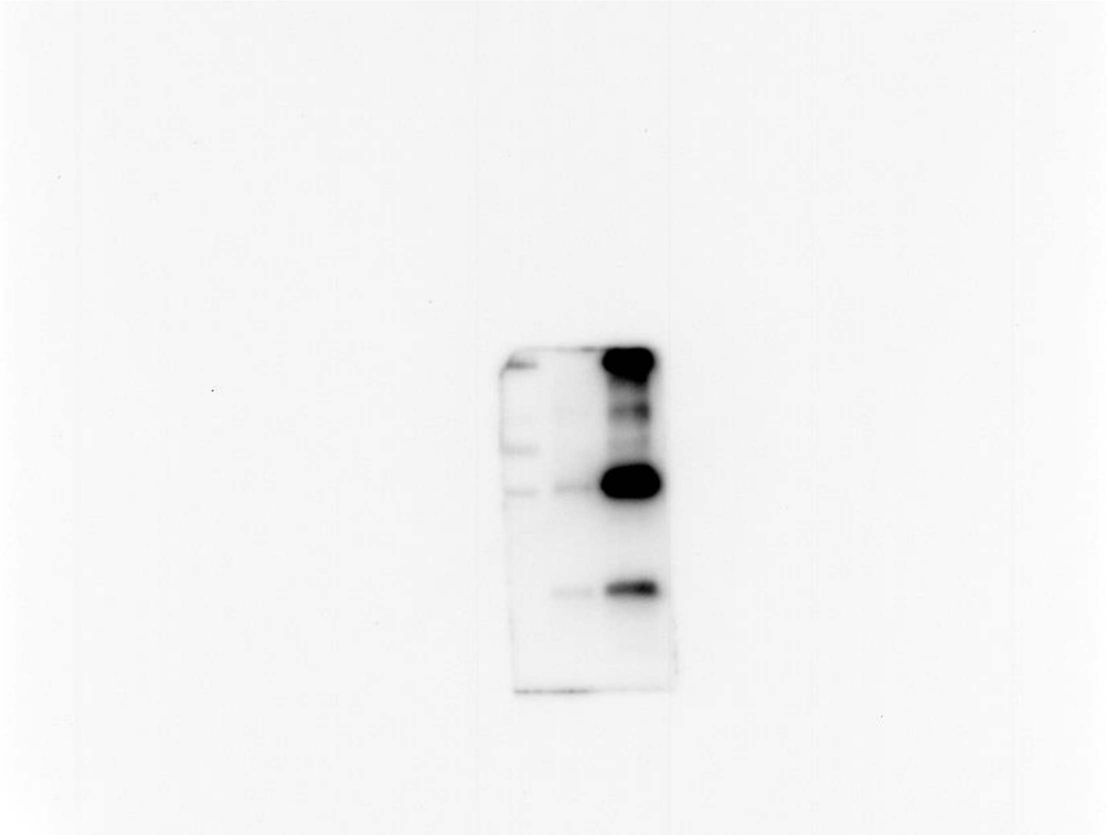

Fig 8C MYC IP FLAG IB

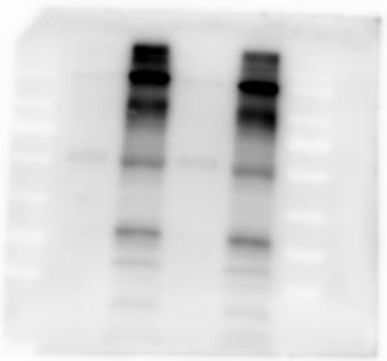

Fig 8D MYC IP UB WB

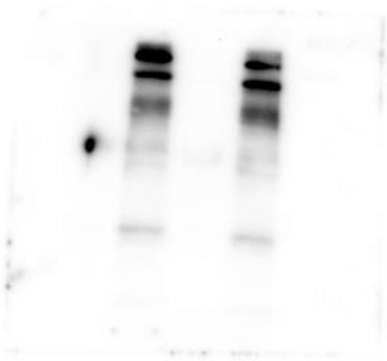

Fig 8D MYC WB

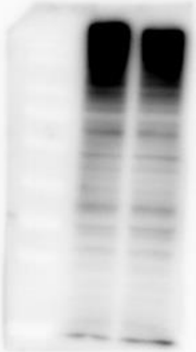

Fig 8D UB input

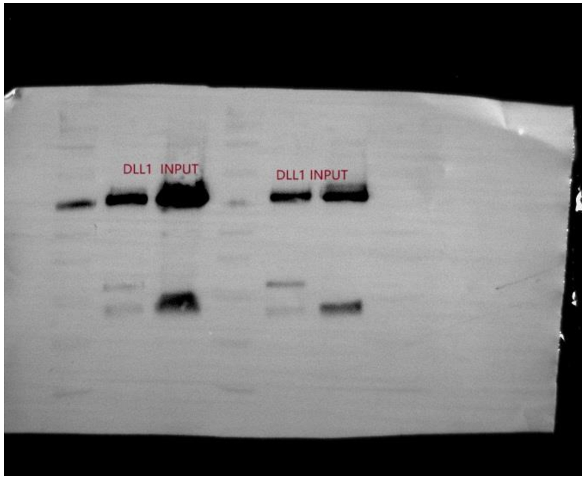

Supplement Fig S15A DLL1 input

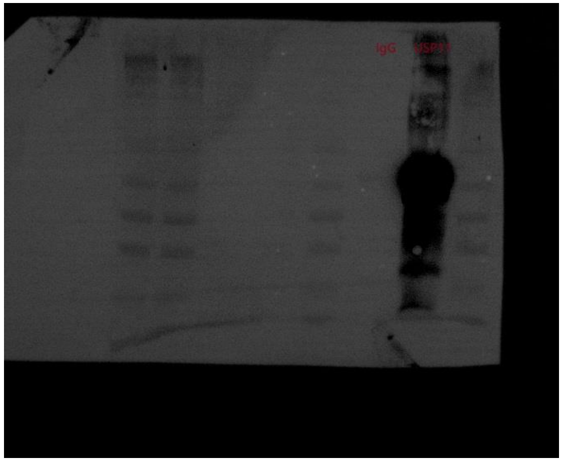

Supplement Fig S15A DLL1 IP Usp11 IB

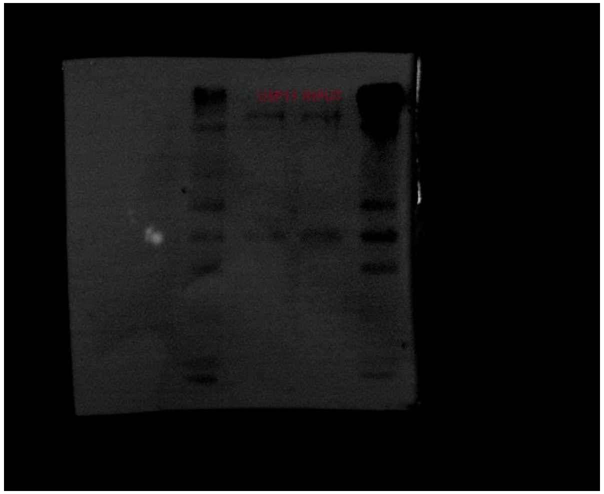

Supplement Fig S15A Usp11 input

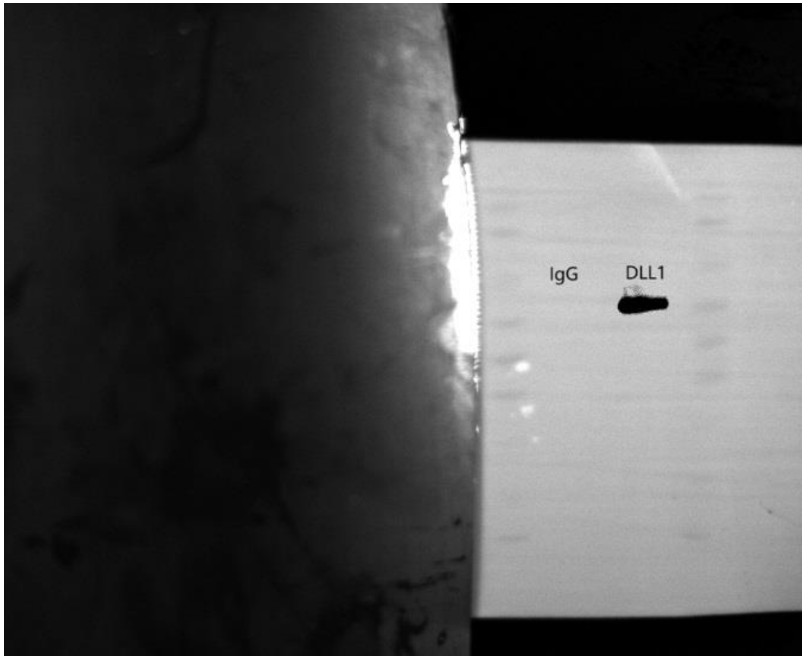

Supplement Fig S15A Usp11 IP DLL1 IB

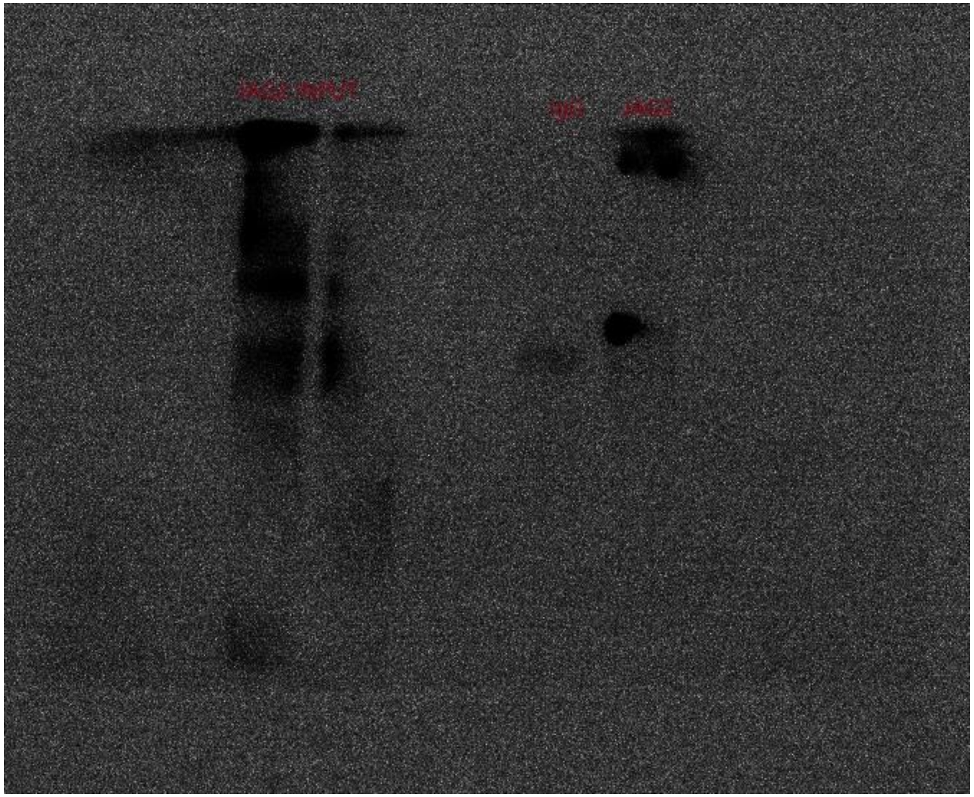

Supplement Fig S15B JAG2 input Usp11 IP JAG2 IB

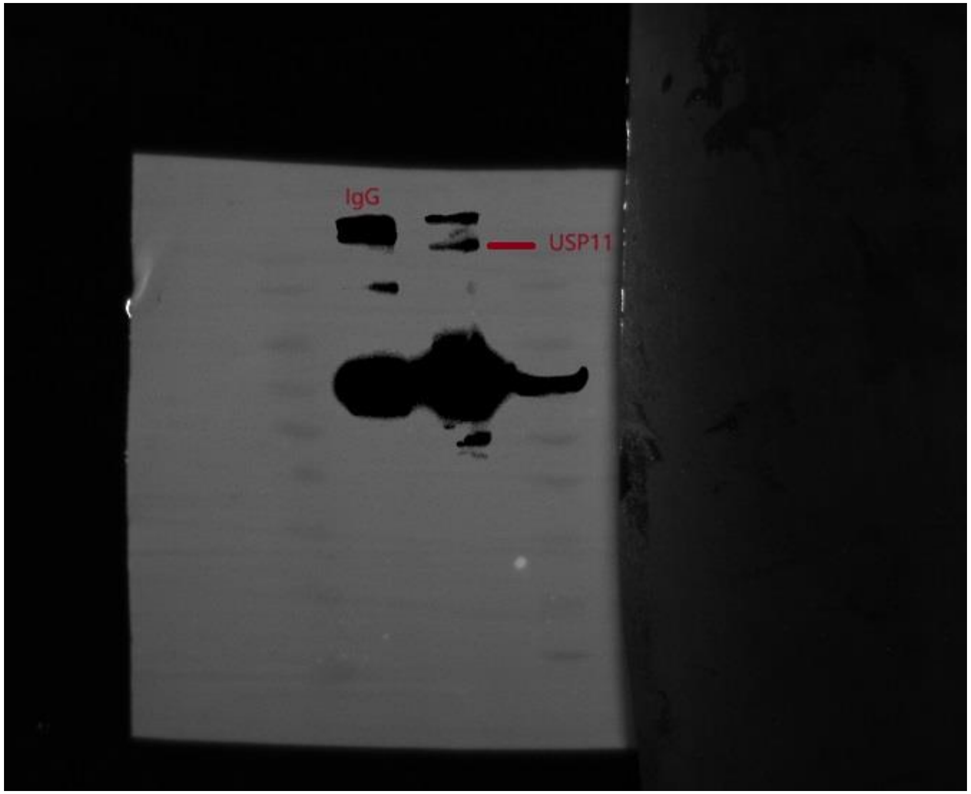

Supplement Fig S15B JAG2 IP Usp11 IB

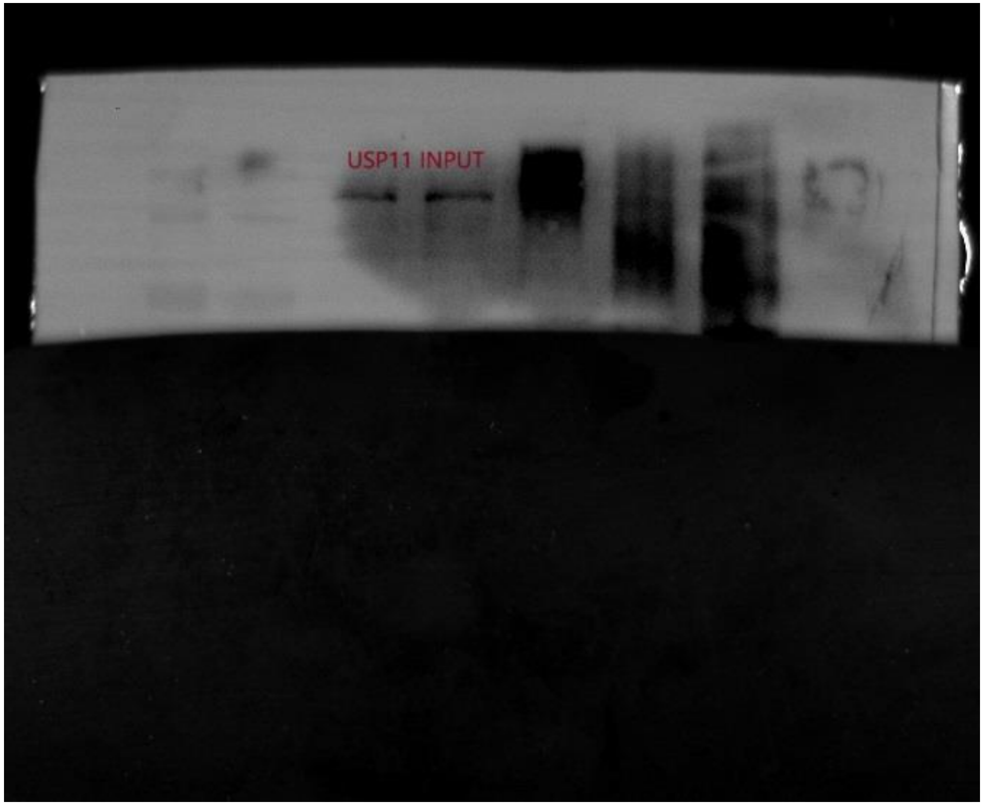

Supplement Fig S15B Usp11 input
